# Ginsenoside Rb1 regulates prefrontal cortical GABAergic transmission in MPTP-treated mice

**DOI:** 10.18632/aging.102095

**Published:** 2019-07-17

**Authors:** Yan Liu, Xiaodan Zong, Jie Huang, Yanfei Guan, Yuanquan Li, Ting Du, Keyin Liu, Xinpan Kang, Chunyan Dou, Xiangdong Sun, Renhua Wu, Lei Wen, Yunlong Zhang

**Affiliations:** 1 Department of Traditional Chinese Medicine, School of Medicine, Xiamen University, Xiamen 361102, China; 2 Key Laboratory of Neuroscience, School of Basic Medical Sciences, Guangzhou Medical University, Guangzhou 511436, China; 3 Department of Medical Imaging, The Second Affiliated Hospital, Medical College of Shantou University, Shantou 515041, China; 4 School of Basic Medical Sciences, Second Affiliated Hospital of Guangzhou Medical University, Guangzhou 510260, China; 5 Provincial Key Laboratory of Medical Molecular Imaging, Shantou 515041, China; 6 Fujian Provincial Key Laboratory of Neurodegenerative Disease and Aging Research, Institute of Neuroscience, School of Medicine, Xiamen University, Xiamen 361102, China; 7 Shenzhen Research Institute of Xiamen University, Shenzhen 518000, China

**Keywords:** Parkinson’s disease, Ginsenoside Rb1, GABAergic transmission, GABA receptors, cognitive impairment

## Abstract

Parkinson’s disease (PD) is a common neurodegenerative disease, featured by motor deficits and non-motor symptoms such as cognitive impairment, and malfunction of gamma-aminobutyric acid (GABA) mediated inhibitory transmission plays an important role in PD pathogenesis. The ginsenoside Rb1 molecule, a major constituent of the extract from the Ginseng root, has been demonstrated to ameliorate motor deficits and prevent dopaminergic neuron death in PD. However, whether Rb1 can regulate GABAergic transmission in PD-associated deficits and its underlying mechanisms are still unclear. In this study, we explored the effects of Rb1 on the GABAergic synaptic transmission in 1-methyl-4-phenyl-1,2,3,6-tetrahydropyridine (MPTP) mouse model of PD. We demonstrated that Rb1 can bind with GABA_A_Rα1 and increase its expression in the SH-SY5Y cells and in the prefrontal cortex (PFC) of MPTP model *in vitro* and *in vivo*. Furthermore, Rb1 can promote prefrontal cortical GABA level and GABAergic transmission in MPTP-treated mice. We also revealed that Rb1 may suppress presynaptic GABA_B_R1 to enhance GABA release and GABA_A_ receptor-mediated inhibitory transmission. In addition, Rb1 attenuated MPTP-induced dysfunctional gait dynamic and cognitive impairment, and this neuroprotective mechanism possibly involved regulating prefrontal cortical GABAergic transmission. Thus, Rb1 may serve as a potential drug candidate for the treatment of PD.

## INTRODUCTION

Parkinson's disease (PD) is a neurological disorder characterized by the classical motor features of parkinsonism and associated with Lewy bodies and loss of dopaminergic neurons in the substantia nigra [1]. Disturbance of the neurotransmitter systems, mainly dopaminergic, glutamatergic, γ-aminobutyric acid (GABA)ergic, is also a hallmark of PD pathology [2]. GABA is the principal inhibitory transmitter in the mammalian brain, and reduced GABA levels were found in the left basal ganglia of PD patients [3]. The level of somatostatin, a marker for a particular GABAergic interneuronal subpopulation, was also found to be significantly decreased in cerebrospinal fluid of PD patients and in GABAergic neurons derived from iPSCs of patients with *parkin* mutations [4, 5]. Malfunction of inhibitory synaptic transmission contributes to PD pathology [6], and it is worthwhile to investigate the underlying mechanism of GABAergic transmission in the PD pathogenesis. Usually, GABA activates two classes of its receptors in the central nervous system (CNS); the ionotropic GABA_A_ receptor channel conducts chloride and bicarbonate ions, while the metabotropic GABA_B_ receptor principally activates the G protein-coupled inwardly rectifying potassium (GIRK) channel [7, 8]. Generally, GABA_A_ receptors mediate fast inhibitory effects and GABA_B_ receptors mediate slow inhibitory effects [9].

Ginseng, the root of *Panax ginseng* C.A. Meyer (Araliaceae), is a widely used herbal medicine for the treatment of neurodegenerative diseases such as Parkinson’s disease and Alzheimer’s disease in the Far East. Several classes of compounds have been isolated from *Panax ginseng*, including ginsenosides, alkaloids, polysaccharides, glucosides, phenolic acid, etc. Among these compounds, ginsenosides are the predominant active constituents of ginseng [10]. To date, more than 70 ginsenosides have been isolated from *Panax ginseng*, among them, ginsenoside Rbl, Rb2, Rc, Rd, Rgl, Rg2, and Re are the major constituents of ginsenosides [10]. Ginsenoside Rb1, the primary active ingredient of *Panax ginseng*, has shown neuroprotective effects in neurodegenerative diseases. Rb1 was reported to protect primary DA neurons and dopaminergic cell lines from 6-OHDA or 1-methyl-4-phenylpyridinium-iodide (MPP^+^) toxicity [11–13]. These neuroprotective effects mainly resulted from Rb1 protecting DA neurons from inflammation activated by nuclear factor kappa B (NF-κB) signaling pathway [14], and inhibiting fibrillation and toxicity of α-synuclein [15]. Our recent study also showed that ginsenoside Rb1 ameliorated motor deficits, prevented DA neuron death, and suppressed α-synuclein expression and astrogliosis in the 1-methyl-4-phenyl-1,2,3,6-tetrahydropyridine (MPTP) mice model of PD [16]. This neuroprotective mechanism involved the regulation of the glutamate transporter GLT-1 to block glutamate excitotoxicity in the nigrostriatal and corticonigral systems [16]. Moreover, Rb1 also actions as neuroprotective agent in other neurodegenerative disease, such as Alzheimer’s disease through its anti-inflammatory and other effects [17, 18]. Whether Rb1 is a promising agent in treating PD and other neurodegenerative disease? What is the potential regulatory mechanism of Rb1 in PD? These are important issues need to be solved.

Though movement dysfunction is the principle manifestation of PD, increasing evidences have indicated that non-motor symptoms are also associated with the progression of PD [19, 20]. Among the non-motor symptoms, progressive cognitive impairment is one of the most common and important features of PD [21]. Notably, working memory deficit is a dominant type of cognitive impairment in PD and about 25% of patients in the early phases of PD experience a decline in their executive functions and working memory [22–24]. Remarkably, prefrontal cortical GABAergic transmission regulates spatial working memory and visual recognition [25, 26], in addition, GABA and glutamate imbalance in the prefrontal cortex (PFC) is associated with the working memory deficiency [27]. Thus, referring to the existing literature indicates a crucial role of GABA in working memory performance, we assume a specific relationship between GABA aberrations and memory impairments in PD. Whether Rb1 regulated GABAergic transmission is correlated with its effects on the cognitve imapirment in PD remain unclear.

In this study, we want to elucidate the effects of Rb1 on the prefrontal cortical GABAergic transmission and the underlying mechanism in PD. Here, we used behaivoral tests to explore the effects of Rb1 on the movement disorder and cognitve imapirment of PD animal model, molecular docking simulation to reveal the interaction between Rb1 and GABA receptors, *in vitro* SH-SY5Y cell culture to indicate the effects of Rb1 on the GABA receptors expression, electrophysiology to record the effects of Rb1 on the synaptic GABA transmission in MPTP model, and GABA-chemical exchange saturation transfer (CEST) magnetic resonance imaging (MRI) to reveal the effects of Rb1 on the prefrontal cortical GABA contents. These findings connect the possible effects of Rb1 in the inhibitory synaptic plasticity of the PFC with memory deficits in PD.

## RESULTS

### Molecular docking analysis of Ginsenoside Rb1 binding with GABA_A_ receptor

**Figure 1:**
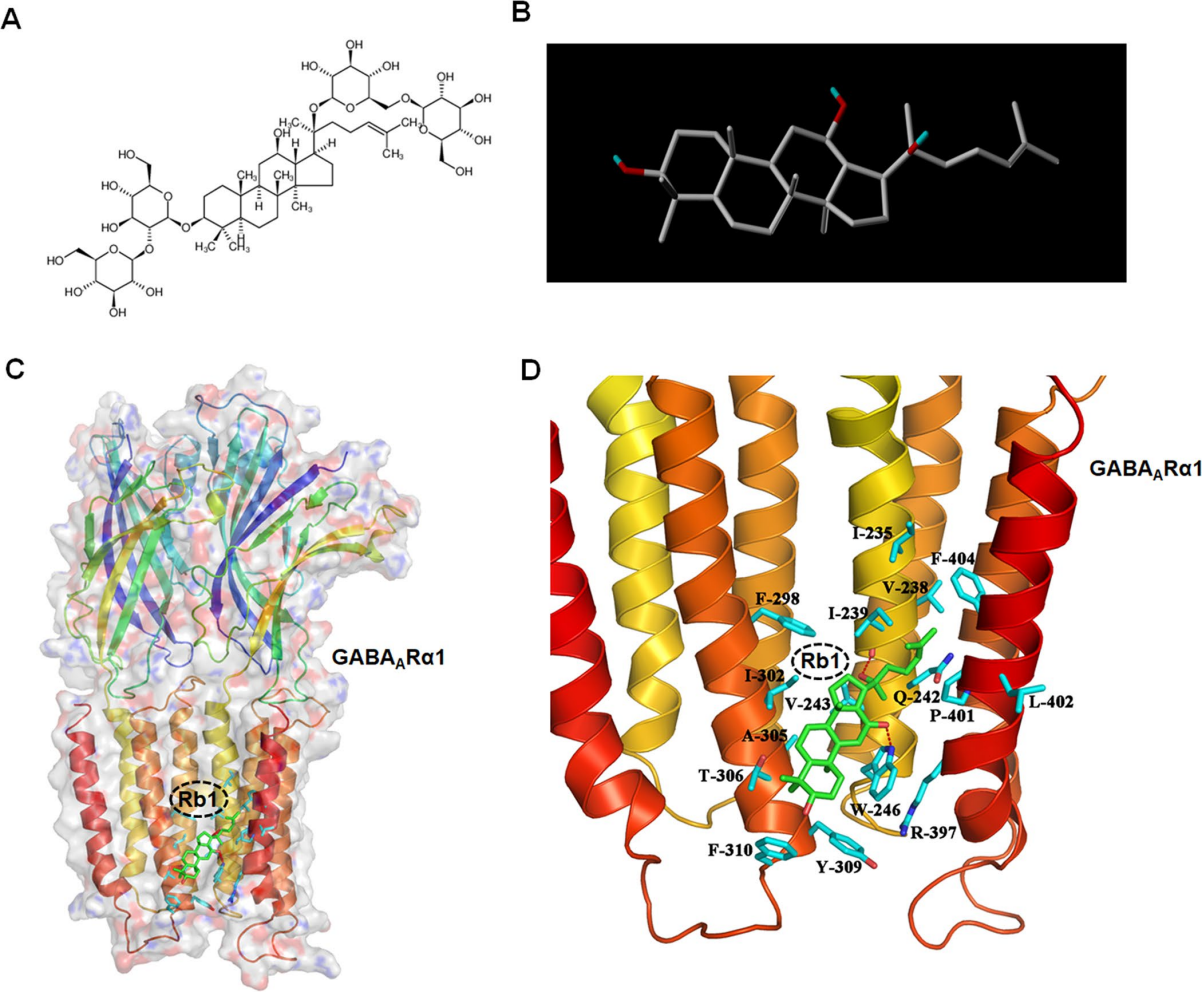
**Molecular docking analysis shows Ginsenoside Rb1 binding with GABA_A_ receptor.** (**A**) Original 2D image of Rb1. (**B**) Modified 3D image of Rb1. (**C**) Overall map of Rb1 interaction with GABA_A_Rα1. (**D**) Interaction between Rb1 with the TMD of GABA_A_Rα1 receptor. Note that when Rb1 (indicted by green stick) was docked in the TMD of GABA_A_Rα1, two hydrogen bonds formed with Ile239 and Trp246 sites (indicated by red dotted line). Besides, Rb1 also formed a hydrophobic interaction with multiple hydrophobic amino acids or hydrophobic parts of polar amino acids in the TMD domain of GABA_A_Rα1 (Ile235, Val238, Ile239, Val243, Trp246, Phe298, Ile302, Ala305, Thr306, Tyr309, Phe310, Ary397, Pro401, Leu402, Phe404) (indicated by blue stick).

Since GABAergic transmission plays an important role in regulating motor and cognitive function in PD, and little is known how Rb1 regulates GABAergic transmission in PD. Firstly, we used Sybyl x-2.1 to evaluate the potential affinity of Rb1 for the transmembrane domain (TMD) of GABA_A_Rα1. The original 2D and modified 3D images of Rb1 were shown in Figure 1A and 1B. The _P_IC50 (-log IC50) for the interaction between Rb1 and TMD of GABA_A_Rα1 was 6.5376. When Rb1 was docked in the TMD of GABA_A_Rα1, two hydrogen bonds formed with Ile239 and Trp246 sites (Figure 1C and 1D). Besides, Rb1 also formed a hydrophobic interaction with multiple hydrophobic amino acids or hydrophobic parts of polar amino acids in the TMD domain of GABA_A_Rα1 (Ile235, Val238, Ile239, Val243, Trp246, Phe298, Ile302, Ala305, Thr306, Tyr309, Phe310, Ary397, Pro401, Leu402, Phe404) (Figure 1C and 1D). Considering the main core structure of Rb1 is hydrophobic, we conclude that Rb1 may interact with the TMD domain of GABA_A_Rα1 via hydrophobic interaction.

### Rb1 upregulates GABA_A_Rα1 expression in SH-SY5Y cell *in vitro*

**Figure 2:**
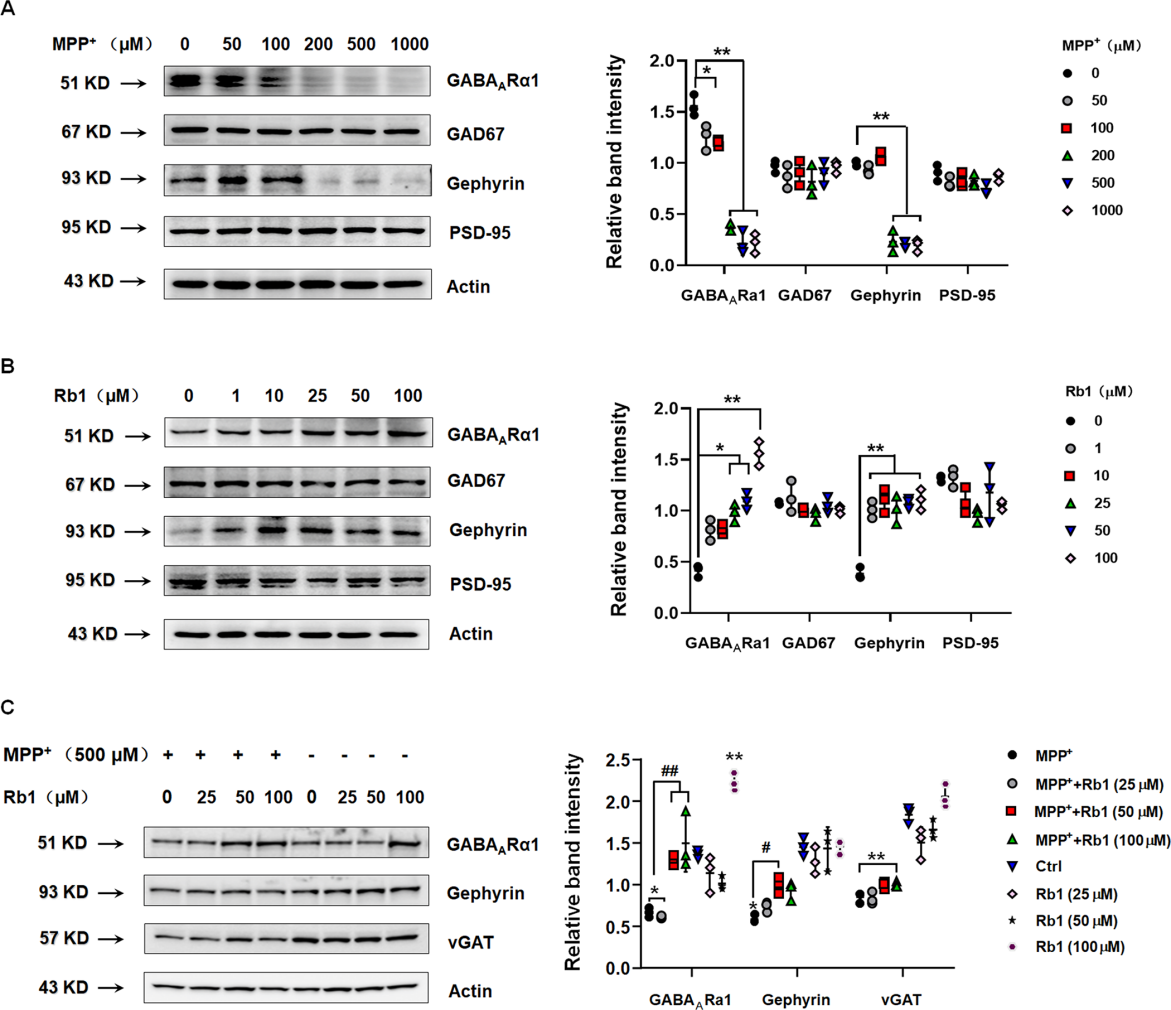
**Rb1 increases GABA_A_Rα1 and gephyrin expression in normal or MPP^+^-treated SH-SY5Y cell.** (**A**) Effect of different concentrations of MPP^+^ on the expression of GABA_A_Rα1, GAD67, gephyrin, and PSD-95 in SH-SY5Y cell. (**B**) Effect of different concentrations of Rb1 on the expression of GABA_A_Rα1, GAD67, gephyrin, and PSD-95 in SH-SY5Y cell. (**C**) Effect of different concentrations of Rb1 on the expression of GABA_A_Rα1, gephyrin, and vGAT in 500 μM MPP^+^-treated SH-SY5Y cell. n = 3. Results are expressed as the mean ± SEM. ^**^P < 0.01 and ^*^P < 0.05 vs. control; ^##^*P* < 0.01 and ^#^*P* < 0.05 vs. MPP^+^ group. Statistical significance was determined by one-way ANOVA and the Bonferroni *post-hoc* test for pairwise comparisons.

Since Rb1 may bind the TMD domain of GABA_A_Rα1, we then examined whether Rb1 can increase GABA_A_Rα1 expression. Using SH-SY5Y cell, we found that 200, 500 and 1000 μM MPP^+^ dramatically decreased the expression of GABA_A_Rα1 and gephyrin, an inhibitory postsynaptic anchoring protein (GABA_A_Rα1: F _5, 12_ = 131.3, *P* < 0.001, post-hoc *P* = 0.0146 for 100 μM MPP^+^, *P* < 0.001 for 200, 500 and 1000 μM MPP^+^; gephyrin: F _5, 12_ = 158.2, *P* < 0.001, post-hoc *P* < 0.001 for 200, 500 and 1000 μM MPP^+^; Figure 2A). While MPP^+^ showed no obvious effects on the glutamic acid decarboxylase 67 (GAD67, a GABA-synthesizing enzyme) and postsynaptic density-95 (PSD-95, an excitatory postsynaptic anchoring protein) (Figure 2A). Moreover, 25, 50 and 100 μM Rb1 increased GABA_A_Rα1 expression and 1, 10, 25, 50 and 100 μM Rb1 increased gephyrin expression in SH-SY5Y cell (GABA_A_Rα1: F _5, 12_ = 58.68, *P* < 0.001, post-hoc *P* = 0.021 for 25 μM Rb1, *P* = 0.0243 for 50 μM Rb1, *P* < 0.001 for 100 μM Rb1; gephyrin: F _5, 12_ = 26.02, *P* < 0.001, post-hoc *P* < 0.001 for 25, 50 and 100 μM Rb1; Figure 2B). Both MPP^+^ and Rb1 showed no obvious effects on the GAD67 and PSD-95 expression (Figure 2B). We further showed that both 50 and 100 μM Rb1 recused GABA_A_Rα1 and 50 μM Rb1 increased gephyrin expression (GABA_A_Rα1: F _7, 16_ = 33.33, *P* < 0.001, post-hoc *P* = 0.0025 for MPP^+^+50 μM Rb1 vs MPP^+^, *P* = 0.0001 for MPP^+^+100 μM Rb1 vs MPP^+^; gephyrin: F _7, 16_ = 20.58, *P* < 0.001, post-hoc *P* = 0.0233 for MPP^+^+50 μM Rb1 vs MPP^+^; Figure 2C), while Rb1 showed no obvious effects on the vesicular GABA transporter (vGAT) expression in MPP^+^-treated SH-SY5Y cell (Figure 2C). Thus, we verified Rb1 can increased GABA_A_Rα1 and postsynaptic gephyrin expression in normal and MPP^+^-treated SH-SY5Y cell.

### Ginsenoside Rb1 mitigates MPTP-induced altered gait parameters in mice

**Figure 3:**
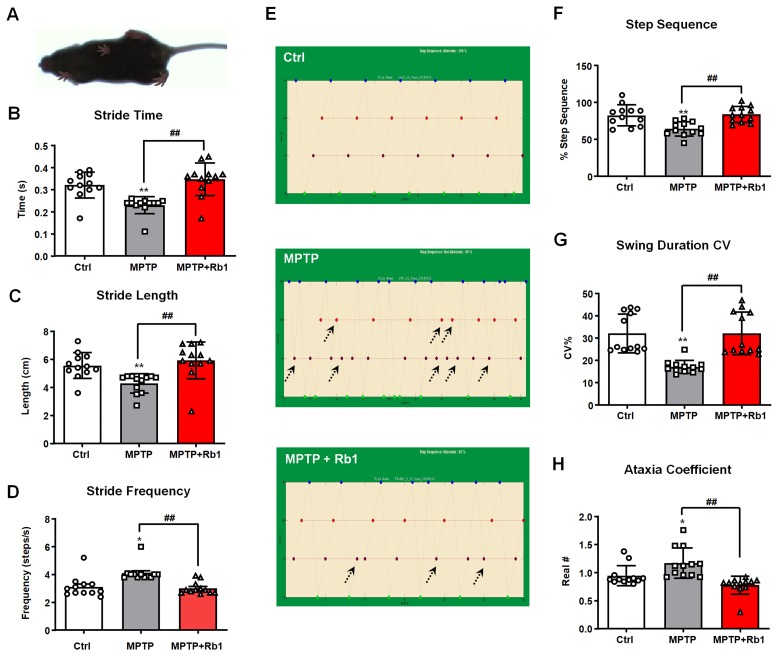
**Rb1 prevented MPTP-induced altered gait dynamics.** (**A**) shows the image extracted from a video recording of the underside of a walking mouse. (**B**–**D**) Effect of Rb1 on the stride time, stride length, and stride frequency in MPTP-treated mice. (**E** and **F**) Effect of Rb1 on the step sequence in MPTP-treated mice. Note that Rb1-prevented MPTP-induced disorder of step sequence as indicated by black dotted arrow in the figure. (**G** and **H**) Effect of Rb1 on the swing duration and ataxia coefficient in MPTP-treated mice. n = 12 per group. Results are expressed as the mean ± SEM. ^**^P < 0.01 and ^*^P < 0.05 vs. control group; ^##^*P* < 0.01 vs. MPTP group. Statistical significance was determined by one-way ANOVA and the Bonferroni *post-hoc* test for pairwise comparisons.

To explore whether Rb1-promoting GABA_A_Rα1 expression showed benefits *in vivo*, we administrated Rb1 in MPTP-induced PD mice model (time-line was shown in Supplementary Figure 1). In our previous study, we have proved that Rb1 can improve movement dysfunction and dopaminergic neuron death in MPTP mice [16], and here we used a gait dynamics test to further confirm the effect of Rb1 on the dyskinesia observed in the MPTP-treated mice. Figure 3A shows an image extracted from the video recording of the ventral side of a mouse walking on the gait-dynamics treadmill belt. Consistent with previous studies, MPTP-treated mice walked with a shorter stride time and stride length, as well as a significantly higher stride frequency. In addition, we showed that MPTP-treated mice that received Rb1 exhibited a more coordinated gait compared with MPTP-treated mice that received saline (stride time: F _2, 33_ = 13.06, *P* < 0.001, post-hoc *P* < 0.001; stride length: F _2, 33_ = 8.765, *P* = 0.0009, post-hoc *P* = 0.001; stride frequency: F _2, 33_ = 11.84, *P* = 0.0001, post-hoc *P* = 0.0003; Figure 3B–3D). Furthermore, administration of Rb1 also increased the step sequence and swing duration of MPTP-treated mice and reduced their ataxia coefficient compared with MPTP-treated that received saline (step sequence: F _2, 33_ = 10.28, *P* = 0.0003, post-hoc *P* = 0.00008; swing duration CV: F _2, 33_ = 15.47, *P* < 0.001, post-hoc *P* < 0.001; ataxia coefficient: F _2, 33_ = 10.85, *P* = 0.0002, post-hoc *P* = 0.0002; Figure 3E–3H). These results are consistent with the effects of Rb1 on other behavioral tests that we reported previously [16].

### Rb1 improved GABAergic transmission in the PFC in MPTP-treated mice

Since we found Rb1 upregulated GABA_A_ receptor and postsynaptic inhibitory protein expression, we then examined whether Rb1 regulates GABAergic transmission in the PFC in MPTP-treated mice by using electrophysiology and GABA-chemical exchange saturation transfer (CEST) magnetic resonance imaging (MRI) (GABA-CEST MRI).

**Figure 4:**
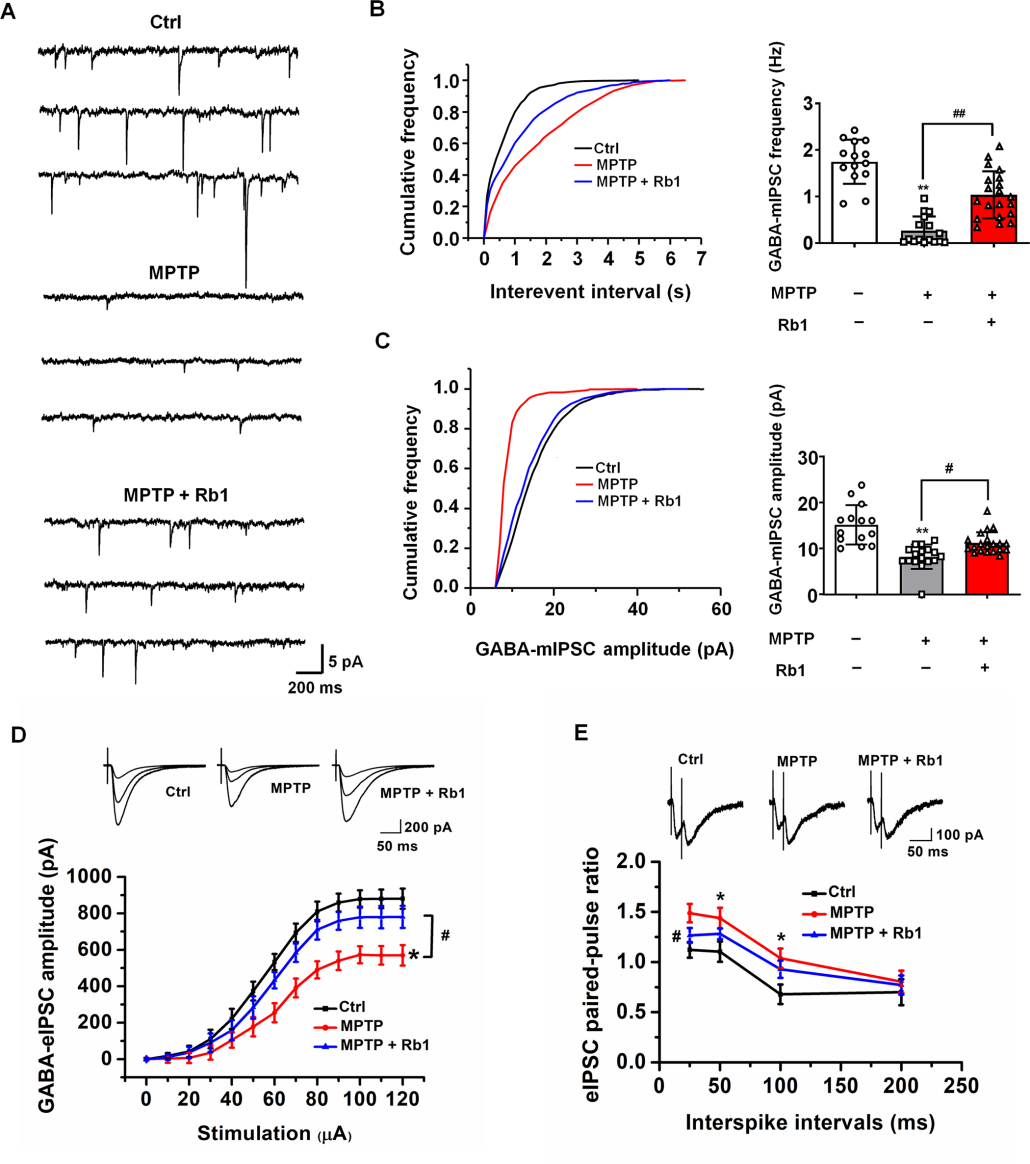
**Rb1 modulates GABAergic transmission in the PFC in MPTP-treated mice.** (**A**) Representative traces of GABA receptor-mediated mIPSCs. All mIPSCs were recorded at a holding potential of −65 mV. (**B**) Cumulative frequency plots of the inter-event interval (left) and quantitative analysis of the frequency of GABA receptor-mediated mIPSCs (right). (**C**) Cumulative frequency plots of mIPSC amplitude (left) and quantitative analysis of the amplitude of GABA receptor-mediated mIPSCs (right). (**D**) Representative traces of eIPSCs at 40, 60, and 100 μA stimulus intensities (top) and stimulus-response curves of PFC neurons from the indicated treatment groups (bottom). (**E**) Paired-pulse ratio analysis. Representative traces (top) and quantification analysis (bottom). Data were obtained from the whole-cell recordings of the prefrontal cortex pyramidal neurons from the three groups of mice including Ctrl mice, MPTP-treated mice, MPTP+Rb1 treated mice. n = 14–20 per group. Results are expressed as the mean ± *SEM*. ^**^P < 0.01 and ^*^P < 0.05 vs. control group; ^##^*p* < 0.01, ^#^*p* < 0.05 vs. MPTP group. Statistical significance was determined by one-way ANOVA and Bonferroni test as *post hoc* comparisons.

The electrophysiological data indicated that MPTP decreased the frequency and amplitude of miniature inhibitory postsynaptic currents (mIPSCs), suggesting that MPTP may decrease the expression and function of postsynaptic GABA receptors (F _2, 47_ = 42.15 and F _2, 47_ = 19.60, both *P* < 0.001, both post-hoc *P* < 0.001 for mIPSC frequency and amplitude, respectively; Figure 4A–4C). Consistent with the results of the mIPSC recordings, the amplitude of evoked inhibitory postsynaptic currents (eIPSCs) was lower in PFC neurons from the MPTP-treated mice compared with the control group (F _2, 48_ = 112.138, *P* < 0.001, post-hoc *P* = 0.014; Figure 4D), confirming the deficits in prefrontal cortical GABAergic synaptic transmission in the MPTP mouse model of PD. Treatment with 10 mg/kg Rb1 increased the frequency (F _2, 47_ = 42.15, *P* < 0.001, post-hoc *P* < 0.001; Figure 4B), and the amplitude of mIPSCs in the PFC of MPTP-treated mice (F _2, 47_ = 19.60, *P* < 0.001, post-hoc *P* = 0.0132; Figure 4C), suggesting that Rb1 may increase the expression and function of GABA receptors or promote postsynaptic GABAergic transmission. The results of the eIPSC recordings also confirmed the protective effects of Rb1 on GABAergic transmission (F _2, 48_ = 112.138, *P* < 0.001, post-hoc *P* = 0.021; Figure 4D). Paired-pulse facilitation was performed to explore whether this was caused by a presynaptic defect, and we found that Rb1 decreased the paired-pulse ratio (PPR) in the MPTP-treated mice at 25 ms (Figure 4E), indicating that the decreased frequencies of currents in the prefrontal cortical neurons may be partially the result of a reduction in the presynaptic GABA release.

**Figure 5:**
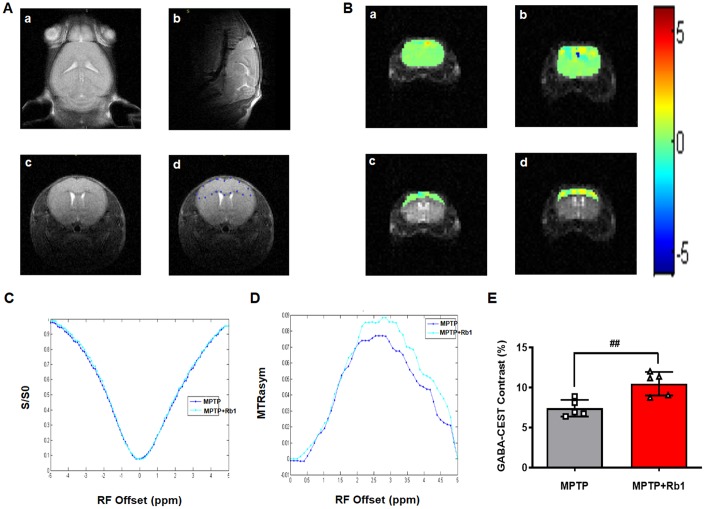
**Rb1 increases GABA level in the PFC in MPTP-treated mice.** (**A**-a, b, and c) Three different imaging orientations of the structural scans of the mouse brain. (**A**-d) The regions of interest (ROIs) in a brain slice positioned in the transverse plane to access the maximum of the PFC. GABA-CEST, B_0_, and B_1_ maps were also acquired from this slice (thickness = 3 mm). (**B**) The top two panels show GABA-CEST maps of the whole brain in (a) the MPTP group and (b) the MPTP+Rb1 group. The bottom two panels show GABA-CEST maps of the ROIs in the PFC of (c) the MPTP group and (d) the MPTP+Rb1 treatment group. (**C** and **D**) Superimposed maps of the Z-spectrum and magnetization transfer ratio asymmetry (MTR_asym_) spectrum between the MPTP group and the MPTP+Rb1 treatment group. (**E**) Quantification of GABA level in the PFC of the MPTP group and the MPTP+Rb1 treatment group. n = 5 per group. Results are expressed as the mean ± SEM. ^##^*P* < 0.01 vs. MPTP group. Statistical significance was determined by a Student's *t*-test.

GABA-CEST MRI was performed to examine the effect of Rb1 on the levels of GABA neurotransmitter in the PFC of MPTP-treated mice. Three-plane orientations and the GABA-CEST maps of the whole brain and the PFC regions of interest (ROIs) in the MPTP and MPTP+Rb1 groups are shown in Figure 5A and 5B. In these two groups, the Z-spectral asymmetry plots obtained from the Z-spectra showed a broad GABA-CEST effect ranging from 1–4 ppm from the bulk water resonance, and the maximum signal intensity effect was at 2.75 ppm (Figure 5C and 5D). The Z-spectral asymmetric images of the MPTP and the MPTP+Rb1 groups showed that the magnetization transfer ratio asymmetry (MTR_asym_) of the MPTP+Rb1 group was higher than that of the MPTP group (Figure 5C and 5D). These results indicated that the level of GABA in the PFC of the MPTP+Rb1 group was significantly higher than that of MPTP group both qualitatively and quantitatively (Student’s *t*-test, df = 8, *P* = 0.005; Figure 5E).

Taken together, these results suggest that Rb1 can rescue the impaired GABAergic transmission and GABA level in the PFC of MPTP-treated mice.

### Rb1 regulates prefrontal cortical GABA_A_ receptor expression in MPTP-treated mice

**Figure 6:**
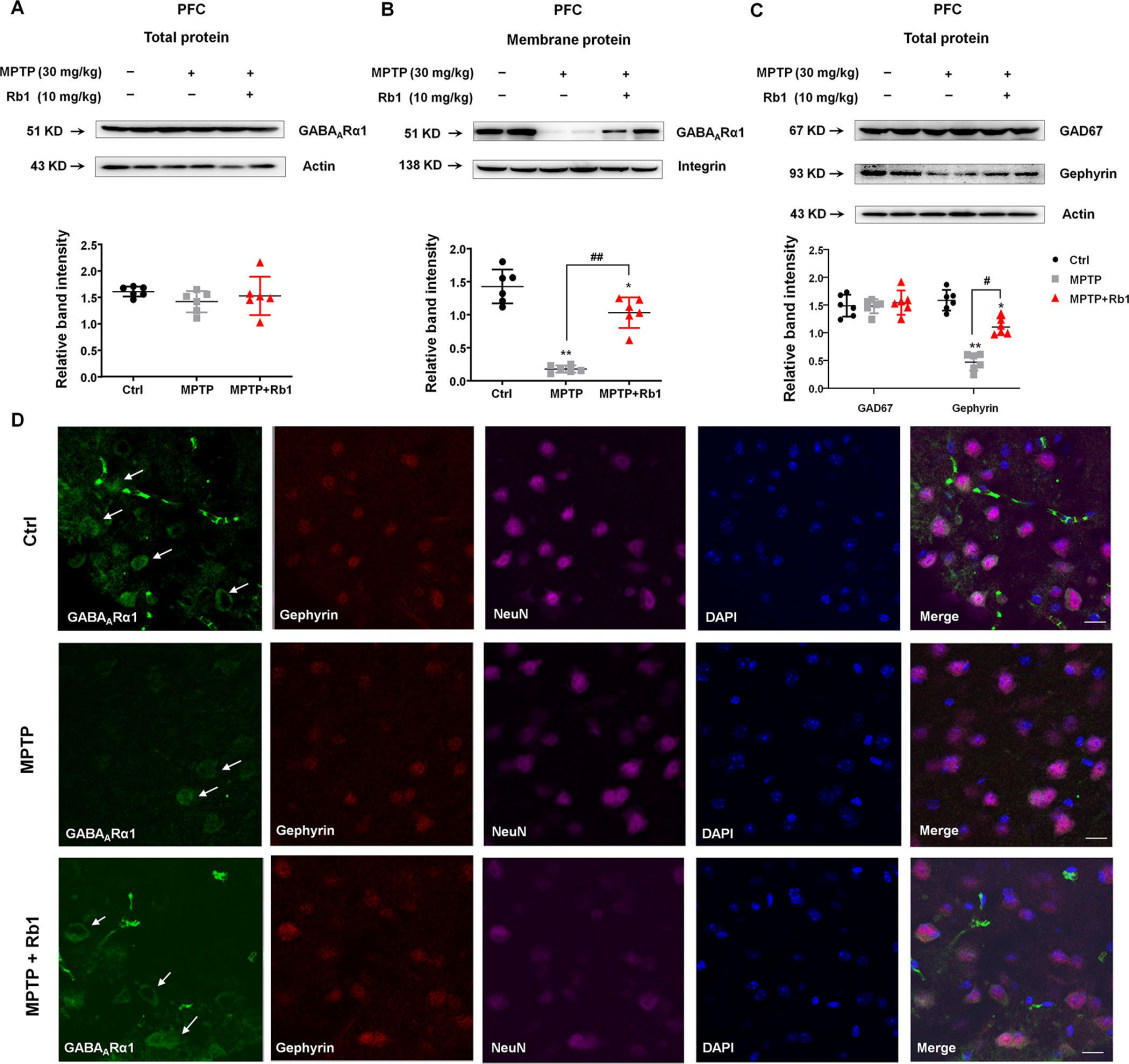
**Rb1 promotes GABA_A_Rα1 receptor expression in the PFC in MPTP-treated mice.** (**A** and **B**) The effect of Rb1 on total GABA_A_Rα1 expression and expression in the membrane fraction of the PFC of MPTP-treated mice was determined by western blotting. (**C**) The effect of Rb1 on GAD67 and gephyrin expression in the PFC of MPTP-treated mice was determined by western blotting. (**D**) Immunofluorescence staining of GABA_A_Rα1 (green), gephyrin (red) and NeuN (purple) in the PFC of MPTP-treated mice. Nuclei are labeled with DAPI (blue). Scale bar = 10 μm. Western blotting results are from two of the six mice in each group and are expressed as the mean ± SEM of three experiments. ^**^P < 0.01 and ^*^P < 0.05 vs. control; ^##^*P* < 0.01 and ^#^*P* < 0.05 vs. MPTP group. Statistical significance was determined by one-way ANOVA and the Bonferroni *post-hoc* test for pairwise comparisons.

Since we found that Rb1 promoted prefrontal cortical GABAergic transmission in the MPTP mouse model, we then investigated whether Rb1 increased GABA_A_ receptor in PFC in MPTP-treated mice. We found that MPTP and Rb1 did not alter the overall expression of GABA_A_Rα1 (Figure 6A), however, MPTP significantly decreased GABA_A_Rα1 expression at the cell membrane (F _2, 15_ = 60.08, *P* < 0.001, post-hoc *P* = 0.0001; Figure 6B). In addition, Rb1 treatment restored the membrane expression of GABA_A_Rα1 (F _2, 15_ = 60.08, *P* < 0.001, post-hoc *P* < 0.001; Figure 6B). Previous studies found that gephyrin stabilized GABA_A_ receptors at the PSD, and postsynaptic gephyrin clusters regulated the surface expression of GABA_A_ receptors [28–30]. Here we reported that Rb1 showed no effects on the GAD67 expression, but it increased gephyrin expression in the MPTP-treated mice (F _2, 15_ = 69.91, *P* < 0.001, post-hoc *P* = 0.016; Figure 6C). In addition, we also found that Rb1 prevented MPTP-induced decrease of GABA_A_Rα1 expression in the PFC (as indicated by the white arrows in Figure 6D). Then we could suggest that Rb1 prevented MPP^+^ or MPTP-decreased GABA_A_Rα1 expression *in vitro* and *in vivo*.

### Rb1 modulates GABA_B_ receptors to enhance GABA_A_ receptor function in MPTP-treated mice

**Figure 7:**
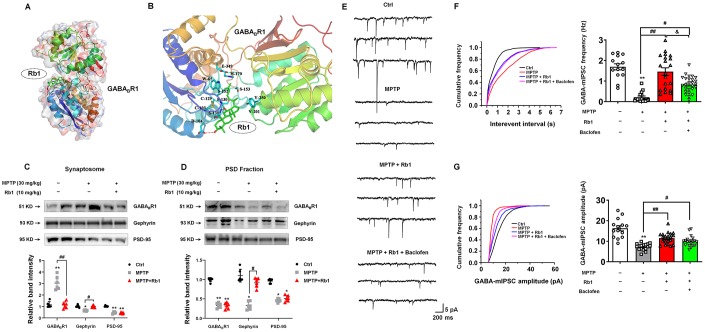
**Rb1 modulates GABA_B_ receptor in the PFC of MPTP-treated mice.** (**A**) Overall map of Rb1 interaction with GABA_B_R1 receptor. (**B**) Interaction between Rb1 with the extracellular domain of 4MS1, agonist conformation of GABA_B_R1 receptor. Note that when Rb1 (indicted by green stick) was docked in the extracellular domain of 4MS1, four hydrogen bonds formed with the amino group of main chain and hydroxyl group of side chain of Ser130 sites, the amino group of main chain of Ser131 site, and the side chain of Asp104 site (indicated by red dotted line). Besides, Rb1 also formed a hydrophobic interaction with multiple hydrophobic amino acids or hydrophobic parts of polar amino acids in the extracellular domain of 4MS1 (Cys103, Cys129, Trp65, His170, Ser153, Tyr250, Val201) (indicated by blue stick). (**C** and **D**) The effect of Rb1 on the GABA_B_ receptor expression in the synaptosome and PSD fraction of the PFC of MPTP-treated mice was determined by western blotting. Western blotting results are from two of the six mice in each group and are expressed as the mean ± SEM of three experiments. (**E**) Representative traces of GABA receptor-mediated mIPSCs in the presence of Rb1 and GABA_B_-receptor agonist Baclofen. All mIPSCs were recorded at a holding potential of −65 mV. (**F**) Cumulative frequency plots of the inter-event interval (left) and quantitative analysis of the frequency of GABA receptor-mediated mIPSCs (right) in the presence of Rb1 and presence of Baclofen. (**G**) Cumulative frequency plots (left) and quantitative analysis (right) of the amplitude of GABA receptor-mediated mIPSCs in the presence of Rb1 and presence of Baclofen. n = 14–20 per group. ^**^P < 0.01 and ^*^P < 0.05 vs. control; ^##^*P* < 0.01 and ^#^*P* < 0.05 vs. MPTP group; ^&^*P* < 0.05 vs. MPTP+Rb1 group. Statistical significance was determined by one-way ANOVA and the Bonferroni *post-hoc* test for pairwise comparisons.

We found that Rb1 increased the frequency of mIPSCs and PPR in the prefrontal cortical neurons in MPTP-treated mice, suggesting Rb1 may also promote presynaptic GABA release in MPTP-treated mice. As we know, presynaptic and postsynaptic GABA_B_ receptors are located on pyramidal neurons in the PFC, and activation of GABA_B_ receptors on presynaptic terminals was shown to reduce glutamate or GABA release at numerous excitatory or inhibitory synapses mainly by inhibiting presynaptic calcium channels [31, 32]. Then we also used Sybyl x-2.1 to evaluate the potential affinity of Rb1 for the binding sites in the extracellular domain of GABA_B_ receptor. A chain of 4MS4 was used for agonist-bound crystal structure of GABA_B_R1 and A chain of 4MS1 was used for antagonist-bound crystal structure of GABA_B_R1. The _P_IC50 (-log IC50) for the interaction between Rb1 and 4MS4 was -50.8634, and for the interaction between Rb1 and 4MS1 was 5.3684, indicating that Rb1 has higher affinity with 4MS1, antagonist conformation of GABA_B_R1. When Rb1 was docked in the extracellular domain of 4MS1, four hydrogen bonds formed with the amino group of main chain and hydroxyl group of side chain of Ser130 sites, the amino group of main chain of Ser131 site, and the side chain of Asp104 site (Figure 7A and 7B). Besides, Rb1 also formed a hydrophobic interaction with multiple hydrophobic amino acids or hydrophobic parts of polar amino acids in the extracellular domain of 4MS1 (Cys103, Cys129, Trp65, His170, Ser153, Tyr250, Val201) (Figure 7A and 7B). The hydrogen bonding interaction together with the hydrophobic interaction maintain Rb1 to stabilize the antagonist conformation of GABA_B_R1, ie, the inactive conformation. The molecular docking results suggest Rb1 may bind with the antagonist conformation of GABA_B_R1.

Since GABA_B_ receptors located in the pre- and post-synaptic synapses, we then examined the effects of Rb1 on the GABA_B_ receptor expression in the synaptosomes and PSD fraction. Notably, we found that Rb1 prevented MPTP-increased GABA_B_R1 expression in the synaptosomes, and showed no obvious effects on the MPP^+^-decreased GABA_B_R1 expression in the PSD fraction in the PFC (synaptosomes: F _2, 15_ = 44.18, *P* < 0.001, post-hoc *P* < 0.001; PSD fraction: F _2, 15_ = 204.2, *P* < 0.001, post-hoc *P* = 0.9501; Figure 7C and 7D). Moreover, Rb1 increased gephyrin expression in both synaptosomes and PSD fraction (synaptosomes: F _2, 15_ = 33.61, *P* < 0.001, post-hoc *P* < 0.001; PSD fraction: F _2, 15_ = 56.33, *P* < 0.001, post-hoc *P* < 0.001; Figure 7C and 7D), consistent with our results in Figure 6C. In addition, MPTP decreased PSD-95 expression in both synaptosomes and PSD fraction and Rb1 showed no obvious effects (Figure 7C and 7D).

We wanted to test whether Rb1’s prevention of the MPTP-increased GABA_B_ receptors contributed to its rescue of GABAergic transmission in MPTP-treated mice. To this end, we used a GABA_B_-receptor agonist to assess the effects of Rb1 on the frequency and amplitude of mIPSCs in MPTP-treated mice (Figure 7E–7G). Here we found that GABA_B_-receptor agonist Baclofen partially decreased Rb1 effects on the frequency of mIPSCs (F _3, 72_ = 15.19, *P* < 0.001, post-hoc *P* = 0.021; Figure 7F), suggesting Rb1-promoted the frequency of mIPSC may be partially due to inhibit presynaptic GABA_B_ receptor expression and function. However, Baclofen showed no obvious effects on the amplitude of mIPSC in the PFC of MPTP+Rb1 group (Figure 7G).

### Ginsenoside Rb1 attenuates memory impairments

Dyskinetic motor symptoms are the characteristic hallmark of PD. However, in addition to such motor symptoms, learning and memory deficits are also associated with PD, and their underlying mechanism is still not well established. Cognitive deficits are a frequent symptom of PD, often occurring in the early stages of disease progression [22–24]. MPTP has been reported to induce deficits in memory performance in primates and rodents [33–36]. In this study, we examined the neuroprotective effects of Rb1 on working memory performance in the MPTP mouse model of PD.

**Figure 8:**
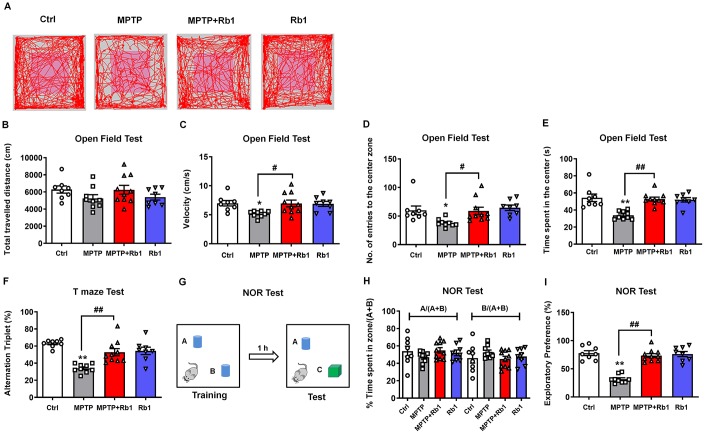
**Rb1 attenuates memory impairments in the MPTP mouse model of PD.** (**A**) Representative path tracings in the open field test. (**B**–**E**) Total travelled distance, movement speed, number of entries to the center, and the time spent in the center of the open-field after Rb1 administration in MPTP-treated mice. (**F**) T-maze results after Rb1 administration in MPTP-treated mice are presented as alternation triplets (consecutive triplets of different arm-choices). (**G**) Design of the novel-object recognition (NOR) task. In the training phase, the mouse is exposed to two objects “A” and “B”. In the testing phase 1 h later, the mouse is exposed to the same object “A” and a novel object “C”. (**H**) Percent time spent with object A or B in the training phase of the NOR test. (**I**) Results from the testing phase of the NOR test show percent time spent with the novel object (exploratory preference). n = 8 in control group, n = 9 in MPTP group, n = 10 in MPTP+Rb1 group and n = 8 in Rb1 group. Results are expressed as the mean ± SEM. ^**^P < 0.01 and ^*^P < 0.05 vs. control group; ^##^*P* < 0.01 and ^#^*P* < 0.05 vs. MPTP group. Statistical significance was determined by one-way ANOVA and the Bonferroni *post-hoc* test for pairwise comparisons.

We conducted open-field to examine the general locomotor activity levels, anxiety, and willingness to explore, and T-maze, novel-object recognition (NOR) tests to examine spontaneous alternation behavior, recognition, and working memory behavior. In the open-field test (Figure 8A–8E), the total distance traveled showed no obvious difference among these four groups (Figure 8B), however, Rb1 prevented MPTP-decreased the movement speed in the open field (F _3, 31_ = 3.911, *P* = 0.0177, post-hoc *P* = 0.032; Figure 8C). In addition, Rb1 prevented MPTP-decreased the number of entries to the center (F _3, 31_ = 4.530, *P* = 0.0096, post-hoc *P* = 0.0423; Figure 8D) and duration in the center (F _3, 31_ = 12.15, *P* < 0.001, post-hoc *P* = 0.0001; Figure 8E). Less time in the center of the open field may also suggest MPTP-treated mice have elevated anxiety levels. To confirm the specific effects of Rb1 on working memory versus anxiety, we performed the elevated plus maze (EPM) test. However, we found that MPTP or Rb1 showed no obvious effects on the total distance, movement speed, number of entries to open arms and the time spent in the open arms (Supplementary Figure 2A–2D), suggesting that Rb1 may influence memory deficiencies rather than anxiety of MPTP-treated mice.

A T-maze test was performed to measure short-term working memory. Consistent with the results of the open-field test, 10 mg/kg Rb1 significantly attenuated the reduced spontaneous alternations caused by MPTP in the T-maze test (F _3, 31_ = 13.67, *P* < 0.001, post-hoc *P* = 0.0011; Figure 8F). Next, we evaluated the effect of Rb1 on working memory impairment in a NOR test in MPTP-treated mice (Figure 8G). In the training session, the mice in all groups spent equal amounts of time exploring each of the two objects (Figure 8H), confirming that there was no biased exploratory preference. However, during the testing session conducted 1 h after the training session, the level of exploratory preference for the novel object in the MPTP-treated mice was significantly decreased, and treatment with 10 mg/kg Rb1 reversed this effect (F _3, 31_ = 36.70, *P* < 0.001, post-hoc *P* = 0.006; Figure 8I). Moreover, MPTP or Rb1 did not affect mice fear expression in the contextual fear memory test (Supplementary Figure 2E and 2F). These results demonstrated that Rb1 ameliorated the impairment of working memory rather than contextual memory in MPTP-treated mice.

## DISCUSSION

As we mentioned above, GABAergic signaling was downregulated in the PD patients and animal models [3–5, 37], and it is urgent to explore the therapeutic strategy targeting GABAergic systems in PD. Here we demonstrated that Rb1 can bind with GABA_A_Rα1 and increase its expression in the PFC of MPTP model. Furthermore, Rb1 can promote prefrontal cortical GABA level and GABAergic transmission in MPTP model. We also indicated that Rb1 may suppress presynaptic GABA_B_R1 to enhance GABA release and GABA_A_ receptor-mediated inhibitory transmission.

### Rb1 promoted GABAergic synaptic transmission in MPTP mice model

Actually, in the past, Rb1 has been proved to modulate GABA_A_ receptor by inhibiting muscimol to the high-affinity site of GABA_A_ receptor [38]. Recently, some groups reported that Ginsenoside compound K (GCK), a main metabolic production of the ginsenoside Rb1, can promote hippocampal GABA release and enhance the GABA_A_ receptor-mediated inhibitory synaptic transmission [39, 40]. These findings suggest Rb1 maybe a key player in the GABAergic transmission in the brain. However, whether Rb1 is an agonist or antagonist of GABA receptor is unclear, the potential binding sites of Rb1 with GABA receptor remain undetermined and whether Rb1 treatment can regulate GABAergic transmission in PD also remain unsolved.

By using molecular docking analysis, for the first time, we identify that Rb1 may be a dual modulator of GABA receptors. We also revealed the potential binding sites of Rb1 with the transmembrane domain of GABA_A_ receptor, and the antagonist domain of GABA_B_ receptor. The hydrophobic interaction supports the binding between Rb1 and GABA_A_ receptor, and hydrogen bond interaction and hydrophobic interaction together maintain Rb1 to stabilize the antagonist conformation of GABA_B_ receptor. We confirmed this molecular docking results by electrophysiology. We found that Rb1 promoted GABA_A_ receptors-mediated mIPSC, eIPSC and PPR in PFC, and the GABA_A_-receptor antagonist bicuculline suppressed nearly all the Rb1-induced GABA_A_ receptor currents (data not shown), suggesting Rb1 increased presynaptic GABA release and GABA actions on the postsynaptic receptors. As we told before, GABA activates the ionotropic GABA_A_ receptor channel to conduct chloride and bicarbonate ions, while GABA activates the metabotropic GABA_B_ receptor principally to induce the GIRK channel [7, 8]. Generally, GABA_A_ receptors mediate fast inhibitory effects and GABA_B_ receptors mediate slow inhibitory effects; hence, mIPSCs are mainly mediated by GABA_A_ receptors. In this work, we also found that the effects of Rb1 on the mIPSC is partially diminished by GABA_B_ receptor agonist Baclofen. It is well established that presynaptic GABA_B_ receptors regulate the release of GABA as autoreceptors, we provide the evidence in Figure 4 to prove that Rb1 can promote the frequency of mIPSC in MPTP mice model, and we use Baclofen to prove the effects of Rb1 on the mIPSC is partially due to inhibiting presynaptic GABA_B_ receptors. While postsynaptic GABA_B_ receptors produce a slow inhibitory postsynaptic potential (IPSP) resulting from the activation of GIRK channels, however, in this study, we recorded the mIPSC current mediated by GABA_A_ receptors, which are fast inhibitory effects, rather than the slow inhibitory IPSP mediated by GABA_B_ receptors. In Figure 6, we have proved that Rb1 increased GABA_A_ receptors, which may explain the effects of Rb1 on the amplitude of mIPSC. However, Baclofen showed no obvious effects on the amplitude of mIPSC, which may be resulted from GABA_B_ receptors have no obvious effects on the inhibitory current mediated by postsynaptic GABA_A_ receptors. Moreover, some groups also reported that Baclofen can reduce the frequency of mIPSC without a change in its amplitude [41, 42]. These results suggest that Rb1 may modulate GABA_B_ receptors via a presynaptic manner, and this effect could be partially abolished by Baclofen. Thus, in this study, Rb1 may exert dual effects on GABA_A_ and GABA_B_ receptors, and Rb1 maintains GABAergic synaptic plasticity in MPTP mice model.

### Rb1 increased GABA_A_ receptors expression in PD cellular and animal model

We also found that Rb1 treatment increased GABA_A_Rα1 expression in MPP^+^-treated SH-SY5Y cell and MPTP-treated mice, consistent with molecular docking and electrophysiological results. As the membrane distribution of GABA_A_ receptors is regulated by their molecular components, which determine postsynaptic targeting and clustering [43, 44]. Increasing evidence indicates that GABA_A_ receptor-associated proteins in the PSD play a role in the dynamic trafficking of GABA_A_ receptors [45, 46]; however, this process is poorly understood in PD. Gephyrin forms clusters at postsynaptic sites and plays an essential role in the postsynaptic clustering of glycine receptors [47, 48]. Similar to the role of excitatory postsynaptic protein PSD-95 in regulating NMDA and AMPA receptor trafficking in the excitatory synapse, gephyrin has been shown to stabilize GABA_A_ receptors at the PSD, and postsynaptic gephyrin regulated the surface expression of GABA_A_ receptors [28–30]. We found that MPP^+^ decreased and Rb1 increased gephyrin expression in SH-SY5Y cell, besides, Rb1 also increased gephyrin expression in MPP^+^-treated SH-SY5Y cell and MPTP-treated mice, suggesting Rb1 may increase gephyrin to stabilize GABA_A_ receptors and improve the inhibitory synaptic plasticity in MPTP mice model. We have to say, in this study, we suggest two possible mechanisms contributing to the Rb1 mediated GABA_A_ receptor expression/stabilization, one is through the direct binding and the other is through gephyrin. However, given that the rescue effect for gephyrin was quite slight in the *in vitro* study of SH-SY5Y cell (Figure 2C), we still need further study to explore the potential effects of Rb1 on the gephyrin in PD.

### Rb1 increased prefrontal cortical GABA content in MPTP mice model

Many endogenous biomolecules can be detected based on their specific chemical structure and exchangeable protons, such as hydroxyl (OH), amide or imino (NH), amine (NH_2_) or thiol (SH) groups. These exchangeable protons can be saturated by pre-saturated radio-frequency pulses and exchanged with the much larger pool of bulk water protons [49–51]. These reductions in water molecule signals can be used to monitor metabolites and reflect their levels in the brain. Since the concentration of some macromolecular solutes is generally small (micromolar or millimolar range), it is difficult to detect the signal on a conventional MRI scan. The continuous CEST signal amplifies the signal from low-concentration solutes, allowing their detection. In this study, we explored the changes in PFC GABA exchange rate in the MPTP-exposed mice before and after Rb1 treatment using CEST MRI. We confirmed that low-concentration GABA and its microenvironmental properties could be measured by this method. This non-invasive technique provides greater sensitivity and spatial resolution compared to the conventional magnetic resonance spectroscopy (^1^H-MRS) techniques [52]. Our GABA-CEST MRI results indicated that the magnetization transfer ratio asymmetry (MTR_asym_) of the MPTP+Rb1 group was higher than that of MPTP group, and GABA level was increased in the PFC by Rb1 treatment in MPTP-exposed mice. We also confirmed that the GABA-CEST signal peak was centered around 2.75 ppm downfield to the bulk water resonance in *in* *vitro* experiments, and the CEST effect increased with concentration.

### Rb1 attenuated cognitive impairment in MPTP mice model

Though DA deficiency of the nigrostriatal pathway accounts for the major motor symptoms of PD, however, PD patients also exhibit impairments in learning and memory, executive function, and visuospatial function, and these cognitive impairments are associated with PFC lesions [53–55]. Working memory deficits are important aspects of the cognitive impairment in PD, particularly in the early stages of PD pathogenesis [22–24]. Since MPTP model has also been reported to successfully mimic the working memory deficit and other cognitive impairments in PD [36, 56–59]. We try to examine the potential link between GABAergic system and cognitive impairment in PD. Although Rb1 has been demonstrated to improve spatial learning and memory by regulating hippocampal neurogenesis or inhibiting neuroinflammation and oxidative stress, little is known about the role of Rb1 in the cognitive impairment of PD. Considering the crucial role of GABA in working memory performance, our findings also suggest the possible link of Rb1-promoted GABAergic transmission with the working memory deficiency in the MPTP mice model.

We firstly examined the locomotor activity, anxiety, and willingness to explore using open-field test. MPTP decreased mice movement speed, suggesting the motor deficits exist in these mice, consistent our previous results. Our EPM test results exclude that MPTP induces anxiety in these mice. Actually, open-field test was also used to examine the willingness to explore. Considering the T-maze and NOR test results, we conclude that MPTP may disrupt the performance of mice to explore in the center. It is widely accepted that prefrontal cortical GABAergic transmission regulates spatial working memory [25, 26]. Optimal GABA neurotransmission in the PFC is thought to be crucial for generating the neural oscillations that underlie working memory [60]. The NOR test we performed in this study is based on the innate propensity of rodents to explore novel objects [74]. This test assesses recognition memory and is thought to be an appropriate model for working memory. Notably, GABA_A_ receptor-mediated GABA transmission was shown to be critical for object recognition [25]. In line with these observations, our data showed that Rb1 ameliorated the deficits in working memory-associated behavioral tests (T-maze test and NOR test) and also regulated GABA_A_ receptor-mediated GABA neurotransmission. These results support a possible link between GABA transmission and working memory in terms of the neuroprotective effects of Rb1 in the MPTP mouse model of PD.

In summary, we demonstrated that Rb1 can promote prefrontal cortical GABA level and GABAergic transmission in MPTP-treated mice. We revealed that Rb1 may exert dual effects on GABA_A_ and GABA_B_ receptors to enhance GABAergic inhibitory transmission. We also suggest Rb1’s effects on GABAergic transmission may be correlated with its’ amelioration in PD-associated motor and cognitive deficits (Supplementary Figure 3). These findings suggest that ginsenoside Rb1 could be a promising treatment for PD.

## MATERIALS AND METHODS

### Reagents

MPTP and MPP^+^ were purchased from Sigma-Aldrich (St. Louis, MO, USA). Ginsenoside Rb1 was purchased from MUST Biotechnology (Chengdu, China). Anti-GABA_A_Rα1 was purchased from Millipore (Bedford, MA, USA). Antibodies for anti-vGAT, anti-integrin, as well as the GABA_B_-receptor agonist Baclofen, were purchased from Santa Cruz Biotechnology (Santa Cruz, CA, USA). Anti-gephyrin, anti-PSD-95, and anti-GABA_B_R1 were purchased from Cell Signaling Technology (Danvers, MA, USA). Anti-GAD67 was purchased from Thermo Scientific (Waltham, MA, USA). Anti-actin was purchased from Beyotime (Shanghai, China). Alexa Fluor 488-conjugated goat anti-mouse, Alexa Fluor 594-conjugated goat anti-rabbit, horseradish peroxidase (HRP)-conjugated goat anti-mouse and anti-rabbit antibodies were purchased from Multi Sciences (Hangzhou, China). EZ-Link Sulfo-NHS-SS-Biotin was purchased from Thermo Scientific (Waltham, MA, USA).

### Molecular docking simulation

Molecular modelling was performed using Sybyl-x 2.1 software from Tripos Inc. (St. Louis, MO). GABA_A_ and GABA_B_ receptor structures used for molecular docking were obtained from the Protein Data Bank (PDB, http://www.rcsb.org; PDB 6CDU for GABA_A_ receptor, 4MS1 and 4MS4 for GABA_B_ receptor). The molecular construction of Rb1 was performed using the "Sketch" module of Sybyl x-2.1, and the 2D structure of Rb1 is shown in Figure 1A.

Ginsenosides, glycosides with steroids or triterpenes as aglycones, are considered to be the major bioactive constituents of ginseng. In the past, lots of evidence indicated that removal of the glycosyl group in ginsenosides (including mild acid hydrolysis, enzymatic conversion, and microbial conversion) is required for improvement of physiological function of ginsenosides [61, 62]. Generally, ginsenosides are metabolized by human intestinal bacteria to deglycosylated forms, which are more easily absorbed in the bloodstream and act as biologically active compounds [63]. Considering this, we removed the di-glycosides of ginsenoside Rb1 in the molecular docking model, and we also added the hydrogen atoms and Gasteiger-Huckel charge to Rb1 (the modified 3D structure of Rb1 is shown in Figure 1B). Finally, the optimization of Rb1 structure was performed by the Sybyl x-2.1 "Powell" energy minimization method. The optimized maximum step size is 1000 steps and the energy gradient is 0.005 kcal/(mol*Å).

For the treatment of GABA_A_ receptor during molecular docking: the AB double strand of crystal structure 6CDU is subjected to hydrogen atomization and AMBER7 FF99 charge treatment. The amino acids surrounding the alphaxalone of the crystal structure 6CDU is selected as the active sites for molecular docking, ie the transmembrane domain (TMD) of GABA_A_ receptor as mentioned previously [64]. Protomol module was used to produce the results for the active sites of molecular docking. For the treatment of GABA_B_ receptor during molecular docking: A strand of crystal structure 4MS1 or 4MS4 is subjected to hydrogen atomization and AMBER7 FF99 charge treatment. The amino acids surrounding the CGP46381 of the crystal structure 4MS1 is selected as the active sites for GABA_B_R1 antagonist molecular docking. The amino acids surrounding the baclofen of the crystal structure 4MS4 is selected as the active sites for GABA_B_R1 agonist molecular docking. The crystal structures of GABA_B_R1 was referred to the previous literature [65]. Protomol module was used to produce the results for the active sites of molecular docking. Surflex-Dock was employed for the molecular docking study, and Surflex-Dock scores (total scores) represent binding affinities.

### Cell culture and drug treatment

SH-SY5Y cell from the American Type Culture Collection (ATCC; Manassas, VA, USA) was cultured in DMEM/F12 (1:1) medium supplemented with FBS (10%, v/v), 100 U/mL of penicillin G, and 100 μg/mL of streptomycin. Cell were maintained at 37 °C in an atmosphere of 5% CO_2_. Stock solutions of MPP^+^ and Rb1 were prepared in 0.01 M phosphate-buffered saline (PBS) and diluted to the appropriate concentrations using cell culture medium. The working concentrations of MPP^+^ used in SH-SY5Y cell were 50, 100, 200, 500, and 1000 μM, and the working concentrations of Rb1 were 1, 10, 25, 50, and 100 μM. MPP^+^ and Rb1 treatment was 24 h in Figure 2. For *in vitro* assays, cells were randomly divided into four groups and treated for 24 h: (1) Control group: Cells were treated with normal culture medium; (2) MPP^+^ group: Cells were treated with culture medium containing 500 μM MPP^+^; (3) MPP^+^+Rb1 group: Cells were treated with culture medium containing 500 μM MPP^+^ and different concentrations of Rb1; (4) Rb1 group: Cells were treated with culture medium containing different concentrations of Rb1.

### Animals

Ten-week-old, male C57BL/6 mice were obtained from SLAC Laboratory Animal Co., Ltd. (Shanghai, China). Three mice were housed per cage with free access to food and water and were subjected to a 12/12-h light/dark cycle (lights on at 6:00 AM) at a constant temperature and humidity. The mice were allowed to adapt to this environment for at least seven days before experiments. All experiments were conducted according to the National Institute of Health guidelines on the care and use of animals (NIH Publications No. 8023, revised 1978) and approved by the Institutional Animal Care and Use Committee of Guangzhou Medical University. Sample sizes were determined based on similar studies with MPTP administration in mice [66].

### Drug treatments

Mice were randomly assigned to three groups: saline+vehicle (Ctrl), MPTP+vehicle (MPTP) and MPTP+Rb1 (10 mg/kg) (MPTP+Rb1). The PD mouse model was generated by administration of MPTP intraperitoneally for five consecutive days at a dose of 30 mg/kg freebase (MPTP-HCl) in saline, as previously described [67, 68]. Three days before the start of MPTP treatment, the ginsenoside Rb1 treatment protocol was commenced. Rb1 was administered intraperitoneally for 14 consecutive days at a dose of 10 mg/kg. The vehicle for MPTP and Rb1 was saline. On days when both drug treatment courses overlapped, the time interval between MPTP and Rb1 injections was more than 12 h. One day after the last Rb1/saline injection, behavioral tests were performed. The experimental timeline was described in Supplementary Figure 1.

### Behavioral tests

The mice were handled for three days before initiation of behavioral tests, and all behavioral tests occurred during the light phase, between 9:00 AM and 4:00 PM.

#### Gait dynamics test

Gait dynamics testing was performed using a motor-driven treadmill with a transparent treadmill belt (DigiGait Imaging System, Mouse Specifics, MA, USA) as previously described [69, 70]. Briefly, mice were placed in an acrylic compartment (L ~25 cm × W ~5 cm), which was mounted on top of the treadmill to maintain the mouse within view of the camera. As the mouse walked on the transparent treadmill belt, digital video images of the ventral plane of the animal were collected at 80 frames per second, and each image represented 12.5 ms. The images were converted to grayscale, and the software automatically provided the gait signals for each of the four limbs by determining the area of each paw as it advanced toward, established contact with, and retreated from the treadmill belt during each stride. The various measurements were defined or calculated as described below: (1) Stride time = the stance duration when the paw of a limb was in contact with the treadmill belt; (2) Stride length = speed / stride frequency; (3) Stride frequency = the number of gait signals over time; (4) Swing duration = the time during which the paw of the same limb was not in contact with the treadmill belt; (5) Swing duration coefficient of variation (CV) = 100 × (swing duration standard deviation / mean swing duration value); (6) Ataxia coefficient = (Maximum stride length – Minimum stride length) / mean stride length.

#### Open-field test

The open field (OF) consisted of a square arena (length [L] 50 cm × width [W] 50 cm) with a white floor and 40-cm walls (height; H). The arena was brightly illuminated and was divided into a central zone (L 25 cm × W 25 cm) and a peripheral zone. Each mouse was placed individually in the central zone, and their locomotion was recorded with a video camera for 5 min and analyzed with Smart 3.0 video tracking software (Panlab, Barcelona, Spain). Behavioral parameters recorded during the 15-min test period included the total distance traveled, the time spent in the central and the peripheral zone. The OF arena was thoroughly cleaned between tests with different animals.

#### T-maze test

This behavioral test was performed on the ninth day after the last MPTP injection, as reported in previous studies [71, 72]. The T-maze was constructed from gray plastic and consisted of three walled arms (L 50 cm × W 10 cm, H 15 cm). Mice were placed at the center of T maze and allowed to move freely through the maze for 8 min. Total distance traveled, time spent in each arm, sequence of arm entries, and total number of arms entered were recorded by Smart 3.0 video tracking software. Percentage of spontaneous alternation was defined as the ratio of consecutive triplets of different arm-choices to the total choices. The maze was thoroughly cleaned between tests with different animals.

#### Novel-object recognition (NOR) test

The NOR test was performed in a Plexiglas open-field box (L 50 cm × W 50 cm × H 40 cm) as described previously [73, 74]. Briefly, the task procedure consisted of three phases: habituation, training (T1), and testing (T2). The habituation phase occurred in the first two days, during which mice were allowed to explore the open field for 5 min. In the T1 phase, two identical objects were placed in the arena, and mice were allowed to explore them for 5 min. The T2 phase began 1 h after the T1 phase; in the T2 phase, one of the objects was replaced with a novel (new) object, and the mouse was allowed to explore for 5 min. The two objects were made of wood and were of similar size, but were different in texture, shape, and color; one object was a blue cylinder and the other was a green cube. The behavior of each mouse during the T2 testing phase was recorded by a video camera, and the total time spent exploring the novel and familiar objects was calculated using Smart 3.0 video tracking software. A recognition index (total time spent with novel object / total time spent exploring both objects) was calculated for each group. The arena and the objects were thoroughly cleaned between tests with different animals.

#### Elevated plus maze (EPM) test

The EPM test was performed according to previously established procedures [37, 68]. Briefly, the plus-shaped maze contained two open arms (L 25 cm × W 5 cm) across from each other and two enclosed arms (L 25 cm × W 5 cm × H 15 cm) across from each other and had an open roof. The maze was set 50 cm above the floor. All measurements were made in a dimly lit experimental room. During a 5-min test period, the following parameters of anxiety-like behavior were recorded: the percentage of entries into the open arms, closed arms, and central platform (reported as the percentage of total entries), and the percentage of time spent in the open arms, closed arms, and central platform (reported as the percentage of 5-min testing time). The test apparatus was thoroughly cleaned between tests with different animals.

#### Fear conditioning test

Contextual fear conditioning was measured in two shock chambers (Chamber A: 25 × 25 × 31 cm, with plastic walls and parallel stainless-steel grid bars in the floor connected to a scrambled shocker; Chamber B: 25 × 25 × 31 cm, with plastic walls and floor) with multiparameter activity monitors (NIR-022MD, The FreezeFrame System, Coulbourn Instruments, Woonsocket, RI, USA). Mice were handled twice a day for three days (each time last longer than 10 min) before the experiments. The conditioned stimulus (CS) used in this study was a 75 dB sound at 2800 Hz, and the unconditioned stimulus (US) was a one time-continuous scrambled foot shock at 0.7 mA for 1 s.

In the training period, mice were placed in the conditioning chamber for 180 s habituation and then subjected to four CS (30 s duration; 80 s intershock interval) that were each terminated with a US (1 s duration). Then mice were removed from the chamber 120 s after the last foot shock. Twenty-four hours later, mice were placed back to the conditioning chamber and monitored for freezing time in 5 min. Forty-eight hours later. Tone-cued fear conditioning was measured in a novel chamber. Each mouse was placed into novel chamber B, monitored for 3 min (pre-tone freezing) and then subjected to 3 min of CS tone exposure (tone-cued freezing). The total time spent freezing in each period was analyzed using FreezeFrame software (SOF-843, IDEO FREEZE).

### GABA-CEST MRI

GABA-CEST MRI was performed as reported in our previous study [75]. Briefly, GABA-CEST MRI was acquired on a 7.0 T small-animal MRI scanner (Agilent Technologies, USA), using a TimeMedical surface coil (TimeMedical Technologies, China). This study tested five MPTP-treated mice and five mice treated with MPTP and Rb1 (MPTP+Rb1). Mice were anesthetized using 1.5–2% isoflurane mixed with O_2_ at a flow rate of 1 L/min and settled into the animal holder inside the volume coil of the MRI scanner. An MRI-compatible small-animal monitoring system (SAII Technologies, USA) was used to monitor respiration and body temperature. Body temperature was maintained at 37°C using air blown through a heater (SAII Technologies, USA).

T_2_-weighted images of three imaging planes were acquired as structural images to position the target plane such that it contained the maximum of the PFC. An improved version of the echo planar imaging (EPI) sequence with a continue wave saturation pulse (B1 = 4.5 μT, pulse width = 3 s) was used to acquire GABA-CEST images with the following parameters: field of view = 25 × 25 mm^2^, shots = 1, slice thickness = 3 mm, matrix size = 64 × 64, flip angle = 15°, time to repetition (TR) = 6000 ms, time to echo (TE) = 27.6 ms, averages = 1. The offset frequency range was set from −5 parts per million (ppm) to 5 ppm (step size = 0.1 ppm) relative to the bulk water resonance frequency, and a total of 103 images were acquired. The time for each Z-spectrum was 337 s.

Image processing and data analysis were performed in MATLAB (MathWorks, version 7.5, R2009b). The Z-spectra and magnetization transfer ratio asymmetry (MTR_asym_) curves were acquired. All the CEST images at different offset frequencies were normalized by a saturation pulse at 10 kHz offset (S_0_). Z-spectra were generated by drawing CEST image intensity as a function of the saturation offset. MTR_asym_ is commonly used to represent the magnitude of the CEST effect and was calculated by the following formula in our CEST analysis

MTR_asym (GABA)_ = 100 × (S_−2.75 ppm_ − S_+2.75 ppm_) / S_0_,

where S_0_ was acquired by saturation frequency offset at 10,000 Hz. S(Δω) and S(−Δω) are values for the same offset frequency in the positive and negative direction, respectively, and S_-2.75 ppm_ and S_+2.75 ppm_ are saturated at -2.75 ppm and +2.75 ppm with respect to water. The radio-frequency field inhomogeneity (B_1_) map in the same layer was acquired to correct the possible inhomogeneity. B_0_ correction was achieved using the water saturation shift referencing (WASSR) method [76, 77].

### Tissue preparation

After the behavioral tests, the mice in each group were euthanized using isoflurane and their tissue used for different assays.

To examine the expression of GABA receptor proteins by western blotting, mice were anesthetized and perfused transcardially with 0.9% saline to remove traces of blood. PFC tissue was collected and stored at −80°C until use.

For morphological experiments, mice were anesthetized and perfused trans-aortically with 0.9% saline followed by fixative (4% paraformaldehyde in 0.01 mol/L PBS, pH 7.4). Then, the fixed brains were removed, stored overnight at 4°C in fixative, and dehydrated in a gradient of 20–30% sucrose. The brain was embedded in optimal cutting temperature (OCT) compound and then cut into sections of 15-μm thickness with a freezing microtome (Leica, Germany) and subsequently stored at −80°C until use.

For electrophysiological analysis, mice were anesthetized with isoflurane, the whole brain was removed, and the experiments were performed as described below.

### Total protein extraction

Brain tissue was homogenized in lysis buffer (Beyotime, Shanghai, China) containing 1 mmol/L phenylmethylsulfonyl fluoride (PMSF), and protein concentrations were determined by a bicinchoninic acid (BCA) assay kit (Beyotime, Shanghai, China). Protein samples were diluted with loading buffer and heated at 95°C for 5 min.

### Cell-surface biotinylation

Cell-surface protein expression was determined using the membrane-impermeable biotinylation reagent EZ-Link Sulfo-NHS-SS-Biotin as described previously [68, 78]. After two washes with ice-cold PBS, brain tissue was incubated with 2.5-mL EZ-Link Sulfo-NHS-SS-Biotin (0.5 mg/mL; dissolved in PBS) at 4°C for two successive 20-min rounds. Then the excess biotin was quenched with 100 mmol/L glycine, and the homogenized tissue was incubated for 20 min in lysis buffer containing a protease inhibitor mixture and 1 mmol/L PMSF. Afterward, the biotinylated proteins were precipitated using agarose-conjugated streptavidin, and the bound proteins were re-solubilized and denatured using loading buffer (Beyotime, Shanghai, China). The resulting protein samples were analyzed by sodium dodecyl sulfate-polyacrylamide gel electrophoresis (SDS-PAGE).

### Postsynaptic density (PSD) protein extraction

Mouse brains were extracted and homogenized on ice in 10 volumes of cold sucrose buffer (0.32 mol/L sucrose, 25 mmol/L HEPES, pH 7.4) with protease and phosphatase inhibitors. The homogenates were centrifuged at 1400 ×*g* for 10 min to separate the supernatant (S1) from the pellet fraction containing cell nuclei and large debris (P1). S1 was saved, and the P1 pellets were resuspended in sucrose buffer and centrifuged at 710 ×*g* for 10 min. The S1 fraction was centrifuged at 10,000 ×*g* for 12 min to separate the supernatant (S2: light membrane and cytosolic fraction) and the pellet (P2: crude synaptosomal fraction). The P2 fraction was washed twice with sucrose buffer and resuspended in cold HEPES-buffered saline (HBS) buffer (25 mmol/L HEPES, pH 7.4, 150 mmol/L NaCl) to obtain the synaptosomal fraction. Finally, the PSD fraction was prepared by solubilizing the synaptosomal fraction in 1% Triton in HBS buffer at 4°C for 30–60 min and centrifuging at 16,000 ×*g* for 20 min to obtain the pellet (synaptic density) and supernatant (synaptic cytosol). The PSD protein samples were analyzed by SDS-PAGE.

### Western blotting

As described previously [68, 78], protein samples were resolved via 12% SDS-PAGE and then transferred to polyvinylidene difluoride (PVDF) membranes. After blocking with 5% bovine serum albumin (BSA) for 2 h at room temperature, the membranes were incubated with primary antibody overnight at 4°C. The next day, the membranes were washed three times in TBS-T (Tris-buffered saline supplemented with 0.1% Tween-20) and incubated with HRP-conjugated goat anti-mouse or anti-rabbit secondary antibodies at room temperature for 1 h. Subsequently, the membranes were washed three additional times with TBS-T. The HRP activity was detected by enhanced chemiluminescence (Millipore, MA, USA), and chemiluminescent immunoreactive bands were visualized using the Tanon imaging system (Shanghai, China). Protein levels were quantified using ImageJ software. Actin or integrin immunoreactivity was used as the control.

### Immunofluorescence

As described previously [78], brain tissue slices were fixed in 4% paraformaldehyde and then rinsed with PBS. Then, the slices were permeabilized with 0.1% Triton X-100 and blocked with 5% BSA. For the multiple staining, the slices were incubated with primary antibodies overnight at 4°C, rinsed with PBS, and then incubated with different fluorescence labeled secondary antibodies for 2 h at 37°C. DAPI was used to stain cell nuclei. Immunostaining was examined using an Olympus FV1000-1X81 laser scanning confocal microscope (Shinjuku, Tokyo, Japan). As a negative control, the primary antibody was replaced with 5% BSA.

### Brain-slice preparation for electrophysiology

As reported in our recent work [16, 79], mice were anesthetized with isoflurane and quickly decapitated. Their brains were rapidly removed and immersed in ice-cold, oxygenated (95% O_2_, 5% CO_2_), sucrose-containing artificial cerebrospinal fluid (ACSF) comprised of (in mmol/L) sucrose (120), NaCl (64), KCl (2.5), NaH_2_PO_4_ (1.25), NaHCO_3_ (26), glucose (10), MgSO_4_ (10), and CaCl_2_ (0.5). Slices of 350-μm thickness were generated using a vibratome (Leica VT1000 S, USA) and then incubated in normal ACSF containing (in mmol/L) NaCl (126), KCl (2.5), NaH_2_PO_4_ (1.25), NaHCO_3_ (26), glucose (10), CaCl_2_ (2), and MgSO_4_ (2). The slices were continuously bubbled with 95% O_2_ and 5% CO_2_ at 32°C for 30 min and then bubbled at room temperature for the duration of the experiment. All experiments were performed within 1–8 h after slice preparation.

### Whole-cell patch-clamp recording

Following incubation, the slices were transferred to a recording chamber in which oxygenated ACSF was warmed to 32°C and superfused over the submerged slices at a flow rate of 2 mL/min. Both mIPSCs and eIPSCs were recorded from PFC pyramidal neurons at a holding potential of −65 mV. For mIPSC recordings, glass pipets were filled with an internal solution containing (in mmol/L) KCl (140), HEPES (10), MgCl_2_ (2), EGTA (0.1), sodium phosphocreatine (10), leupeptin (0.2), Mg-ATP (4), and Na-GTP (0.3) at pH 7.3 and 290 mOsm. For recordings of GABA_A_ receptor-mediated mIPSCs, AMPA and NMDA glutamate receptors were pharmacologically blocked using 20 μmol/L 6-cyano-7-nitroquinoxaline-2,3-dione (CNQX) and 50 μmol/L DL-2-amino-5-phosphonovaleric acid (APV). Tetrodotoxin (1 μmol/L) was included in the perfusion solution for mIPSC recordings. To explore the effect of GABA_B_ receptor activation on inhibitory transmission, a GABA_B_-receptor agonist Baclofen (100 μmol/L) was added to the perfusion ACSF during the mIPSC recordings. For eIPSC recordings, the perfusion ACSF was supplemented with 50 μmol/L APV and 20 μM CNQX to block NMDA and AMPA receptors, respectively. The recording electrodes were positioned on PFC pyramidal neurons in cortical layer II–IV, and the stimulating electrodes were placed in layer Ι. Neurons were allowed to equilibrate in the recording chamber with the perfusion ACSF for at least 5 min before recording. The eIPSCs was generated with a concentric bipolar stimulating electrode (FHC, Inc.) positioned 300 μm from the pyramidal neuron being recorded, and single pulses of 0.1 ms were delivered at 0.1 Hz. The stimulus intensity used to generate eIPSCs was 60 μA. Data were acquired with a MultiClamp 700B amplifier (Molecular Devices, Sunnyvale, CA) at 10 kHz, filtered at 1 kHz, and saved for further analysis using pClamp software (Molecular Devices). Only recordings in which the access resistance changed less than 15% were retained for analysis. mIPSCs were analyzed with Mini Analysis software (Synaptosoft, Inc.).

### Statistical analysis

Statistical analysis of the data was performed on SPSS 16.0 (SPSS Inc., Chicago, IL) using one-way analysis of variance (ANOVA) followed by the Bonferroni post-hoc test for multiple comparisons and the Student's *t*-test for comparisons between two groups. All data are expressed as the mean ± standard error of the mean (SEM), and the statistical significance level was set at *P* < 0.05.

## Supplementary Material

Supplementary Figures

## References

[1] Goetz CG. The history of Parkinson’s disease: early clinical descriptions and neurological therapies. Cold Spring Harb Perspect Med. 2011; 1:a008862. 10.1101/cshperspect.a008862 22229124 PMC3234454

[2] Sanjari Moghaddam H, Zare-Shahabadi A, Rahmani F, Rezaei N. Neurotransmission systems in Parkinson’s disease. Rev Neurosci. 2017; 28:509–36. 10.1515/revneuro-2016-0068 28328536

[3] Elmaki EE, Gong T, Nkonika DM, Wang G. Examining alterations in GABA concentrations in the basal ganglia of patients with Parkinson’s disease using MEGA-PRESS MRS. Jpn J Radiol. 2018; 36:194–99. 10.1007/s11604-017-0714-z 29280067

[4] Dupont E, Christensen SE, Hansen AP, de Fine Olivarius B, Orskov H. Low cerebrospinal fluid somatostatin in Parkinson disease: an irreversible abnormality. Neurology. 1982; 32:312–14. 10.1212/WNL.32.3.312 6121303

[5] Iwasawa C, Kuzumaki N, Suda Y, Kagawa R, Oka Y, Hattori N, Okano H, Narita M. Reduced expression of somatostatin in GABAergic interneurons derived from induced pluripotent stem cells of patients with parkin mutations. Mol Brain. 2019; 12:5. 10.1186/s13041-019-0426-7 30658665 PMC6339354

[6] Milosevic L, Gramer R, Kim TH, Algarni M, Fasano A, Kalia SK, Hodaie M, Lozano AM, Popovic MR, Hutchison WD. Modulation of inhibitory plasticity in basal ganglia output nuclei of patients with Parkinson’s disease. Neurobiol Dis. 2019; 124:46–56. 10.1016/j.nbd.2018.10.020 30391540

[7] Bormann J. Electrophysiology of GABAA and GABAB receptor subtypes. Trends Neurosci. 1988; 11:112–16. 10.1016/0166-2236(88)90156-7 2465608

[8] Andrade R, Malenka RC, Nicoll RA. A G protein couples serotonin and GABAB receptors to the same channels in hippocampus. Science. 1986; 234:1261–65. 10.1126/science.2430334 2430334

[9] Kapur A, Pearce RA, Lytton WW, Haberly LB. GABAA-mediated IPSCs in piriform cortex have fast and slow components with different properties and locations on pyramidal cells. J Neurophysiol. 1997; 78:2531–45. 10.1152/jn.1997.78.5.2531 9356403

[10] Ru W, Wang D, Xu Y, He X, Sun YE, Qian L, Zhou X, Qin Y. Chemical constituents and bioactivities of Panax ginseng (C. A. Mey.). Drug Discov Ther. 2015; 9:23–32. 10.5582/ddt.2015.01004 25788049

[11] Ahmed T, Raza SH, Maryam A, Setzer WN, Braidy N, Nabavi SF, de Oliveira MR, Nabavi SM. Ginsenoside Rb1 as a neuroprotective agent: A review. Brain Res Bull. 2016; 125:30–43. 10.1016/j.brainresbull.2016.04.002 27060612

[12] Radad K, Gille G, Moldzio R, Saito H, Ishige K, Rausch WD. Ginsenosides Rb1 and Rg1 effects on survival and neurite growth of MPP+-affected mesencephalic dopaminergic cells. J Neural Transm (Vienna). 2004; 111:37–45. 10.1007/s00702-003-0063-1 14714214

[13] González-Burgos E, Fernandez-Moriano C, Gómez-Serranillos MP. Potential neuroprotective activity of Ginseng in Parkinson’s disease: a review. J Neuroimmune Pharmacol. 2015; 10:14–29. 10.1007/s11481-014-9569-6 25349145

[14] Li DW, Zhou FZ, Sun XC, Li SC, Yang JB, Sun HH, Wang AH. Ginsenoside Rb1 protects dopaminergic neurons from inflammatory injury induced by intranigral lipopolysaccharide injection. Neural Regen Res. 2019; 14:1814–22. 10.4103/1673-5374.257536 31169200 PMC6585553

[15] Ardah MT, Paleologou KE, Lv G, Menon SA, Abul Khair SB, Lu JH, Safieh-Garabedian B, Al-Hayani AA, Eliezer D, Li M, El-Agnaf OM. Ginsenoside Rb1 inhibits fibrillation and toxicity of alpha-synuclein and disaggregates preformed fibrils. Neurobiol Dis. 2015; 74:89–101. 10.1016/j.nbd.2014.11.007 25449909 PMC4882765

[16] Zhang YL, Liu Y, Kang XP, Dou CY, Zhuo RG, Huang SQ, Peng L, Wen L. Ginsenoside Rb1 confers neuroprotection via promotion of glutamate transporters in a mouse model of Parkinson’s disease. Neuropharmacology. 2018; 131:223–37. 10.1016/j.neuropharm.2017.12.012 29241654

[17] Lin J, Gao S, Wang T, Shen Y, Yang W, Li Y, Hu H. Ginsenoside Rb1 improves learning and memory ability through its anti-inflammatory effect in Aβ_1-40_ induced Alzheimer’s disease of rats. Am J Transl Res. 2019; 11:2955–68. 31217866 PMC6556649

[18] Zhao J, Lu S, Yu H, Duan S, Zhao J. Baicalin and ginsenoside Rb1 promote the proliferation and differentiation of neural stem cells in Alzheimer’s disease model rats. Brain Res. 2018; 1678:187–94. 10.1016/j.brainres.2017.10.003 29038007

[19] Chaudhuri KR, Healy DG, Schapira AH, and National Institute for Clinical Excellence. Non-motor symptoms of Parkinson’s disease: diagnosis and management. Lancet Neurol. 2006; 5:235–45. 10.1016/S1474-4422(06)70373-8 16488379

[20] Kalia LV, Lang AE. Parkinson’s disease. Lancet. 2015; 386:896–912. 10.1016/S0140-6736(14)61393-3 25904081

[21] Svenningsson P, Westman E, Ballard C, Aarsland D. Cognitive impairment in patients with Parkinson’s disease: diagnosis, biomarkers, and treatment. Lancet Neurol. 2012; 11:697–707. 10.1016/S1474-4422(12)70152-7 22814541

[22] Foltynie T, Brayne CE, Robbins TW, Barker RA. The cognitive ability of an incident cohort of Parkinson’s patients in the UK. The CamPaIGN study. Brain. 2004; 127:550–60. 10.1093/brain/awh067 14691062

[23] Riedel O, Klotsche J, Spottke A, Deuschl G, Förstl H, Henn F, Heuser I, Oertel W, Reichmann H, Riederer P, Trenkwalder C, Dodel R, Wittchen HU. Cognitive impairment in 873 patients with idiopathic Parkinson’s disease. Results from the German Study on Epidemiology of Parkinson’s Disease with Dementia (GEPAD). J Neurol. 2008; 255:255–64. 10.1007/s00415-008-0720-2 18204803

[24] Seubert-Ravelo AN, Yáñez-Téllez MG, Salgado-Ceballos H, Escartín-Pérez RE, Neri-Nani GA, Velázquez-Osuna S. Mild Cognitive Impairment in Patients with Early-Onset Parkinson’s Disease. Dement Geriatr Cogn Disord. 2016; 42:17–30. 10.1159/000447533 27467581

[25] Damgaard T, Plath N, Neill JC, Hansen SL. Extrasynaptic GABAA receptor activation reverses recognition memory deficits in an animal model of schizophrenia. Psychopharmacology (Berl). 2011; 214:403–13. 10.1007/s00213-010-2039-9 20957350

[26] Luo P, Chen C, Lu Y, Fu T, Lu Q, Xu X, Li C, He Z, Guo L. Baclofen ameliorates spatial working memory impairments induced by chronic cerebral hypoperfusion via up-regulation of HCN2 expression in the PFC in rats. Behav Brain Res. 2016; 308:6–13. 10.1016/j.bbr.2016.04.020 27085590

[27] Marsman A, Mandl RC, Klomp DW, Cahn W, Kahn RS, Luijten PR, Hulshoff Pol HE. Intelligence and Brain Efficiency: Investigating the Association between Working Memory Performance, Glutamate, and GABA. Front Psychiatry. 2017; 8:154. 10.3389/fpsyt.2017.00154 28966597 PMC5605555

[28] Dejanovic B, Semtner M, Ebert S, Lamkemeyer T, Neuser F, Lüscher B, Meier JC, Schwarz G. Palmitoylation of gephyrin controls receptor clustering and plasticity of GABAergic synapses. PLoS Biol. 2014; 12:e1001908. 10.1371/journal.pbio.1001908 25025157 PMC4099074

[29] Fritschy JM, Harvey RJ, Schwarz G. Gephyrin: where do we stand, where do we go? Trends Neurosci. 2008; 31:257–64. 10.1016/j.tins.2008.02.006 18403029

[30] Tyagarajan SK, Ghosh H, Yévenes GE, Imanishi SY, Zeilhofer HU, Gerrits B, Fritschy JM. Extracellular signal-regulated kinase and glycogen synthase kinase 3β regulate gephyrin postsynaptic aggregation and GABAergic synaptic function in a calpain-dependent mechanism. J Biol Chem. 2013; 288:9634–47. 10.1074/jbc.M112.442616 23408424 PMC3617267

[31] Doze VA, Cohen GA, Madison DV. Calcium channel involvement in GABAB receptor-mediated inhibition of GABA release in area CA1 of the rat hippocampus. J Neurophysiol. 1995; 74:43–53. 10.1152/jn.1995.74.1.43 7472344

[32] Kolaj M, Bai D, Renaud LP. GABAB receptor modulation of rapid inhibitory and excitatory neurotransmission from subfornical organ and other afferents to median preoptic nucleus neurons. J Neurophysiol. 2004; 92:111–22. 10.1152/jn.00014.2004 14973311

[33] Ko WK, Camus SM, Li Q, Yang J, McGuire S, Pioli EY, Bezard E. An evaluation of istradefylline treatment on Parkinsonian motor and cognitive deficits in 1-methyl-4-phenyl-1,2,3,6-tetrahydropyridine (MPTP)-treated macaque models. Neuropharmacology. 2016; 110:48–58. 10.1016/j.neuropharm.2016.07.012 27424102

[34] Ho SC, Hsu CC, Pawlak CR, Tikhonova MA, Lai TJ, Amstislavskaya TG, Ho YJ. Effects of ceftriaxone on the behavioral and neuronal changes in an MPTP-induced Parkinson’s disease rat model. Behav Brain Res. 2014; 268:177–84. 10.1016/j.bbr.2014.04.022 24755306

[35] Decamp E, Tinker JP, Schneider JS. Attentional cueing reverses deficits in spatial working memory task performance in chronic low dose MPTP-treated monkeys. Behav Brain Res. 2004; 152:259–62. 10.1016/j.bbr.2003.10.007 15196793

[36] Miyoshi E, Wietzikoski S, Camplessei M, Silveira R, Takahashi RN, Da Cunha C. Impaired learning in a spatial working memory version and in a cued version of the water maze in rats with MPTP-induced mesencephalic dopaminergic lesions. Brain Res Bull. 2002; 58:41–47. 10.1016/S0361-9230(02)00754-2 12121811

[37] Lozovaya N, Eftekhari S, Cloarec R, Gouty-Colomer LA, Dufour A, Riffault B, Billon-Grand M, Pons-Bennaceur A, Oumar N, Burnashev N, Ben-Ari Y, Hammond C. GABAergic inhibition in dual-transmission cholinergic and GABAergic striatal interneurons is abolished in Parkinson disease. Nat Commun. 2018; 9:1422. 10.1038/s41467-018-03802-y 29651049 PMC5897332

[38] Kimura T, Saunders PA, Kim HS, Rheu HM, Oh KW, Ho IK. Interactions of ginsenosides with ligand-bindings of GABA(A) and GABA(B) receptors. Gen Pharmacol. 1994; 25:193–99. 10.1016/0306-3623(94)90032-9 8026706

[39] Zeng X, Hu K, Chen L, Zhou L, Luo W, Li C, Zong W, Chen S, Gao Q, Zeng G, Jiang D, Li X, Zhou H, Ouyang DS. The Effects of Ginsenoside Compound K Against Epilepsy by Enhancing the γ-Aminobutyric Acid Signaling Pathway. Front Pharmacol. 2018; 9:1020. 10.3389/fphar.2018.01020 30254585 PMC6142013

[40] Bae MY, Cho JH, Choi IS, Park HM, Lee MG, Kim DH, Jang IS. Compound K, a metabolite of ginsenosides, facilitates spontaneous GABA release onto CA3 pyramidal neurons. J Neurochem. 2010; 114:1085–96. 10.1111/j.1471-4159.2010.06833.x 20524959

[41] Lu Y, Burger RM, Rubel EW. GABA(B) receptor activation modulates GABA(A) receptor-mediated inhibition in chicken nucleus magnocellularis neurons. J Neurophysiol. 2005; 93:1429–38. 10.1152/jn.00786.2004 15483063

[42] Iyadomi M, Iyadomi I, Kumamoto E, Tomokuni K, Yoshimura M. Presynaptic inhibition by baclofen of miniature EPSCs and IPSCs in substantia gelatinosa neurons of the adult rat spinal dorsal horn. Pain. 2000; 85:385–93. 10.1016/S0304-3959(99)00285-7 10781911

[43] Lorenz-Guertin JM, Jacob TC. GABA type a receptor trafficking and the architecture of synaptic inhibition. Dev Neurobiol. 2018; 78:238–270. 10.1002/dneu.22536 28901728 PMC6589839

[44] Mele M, Leal G, Duarte CB. Role of GABA_A_ R trafficking in the plasticity of inhibitory synapses. J Neurochem. 2016; 139:997–1018. 10.1111/jnc.13742 27424566

[45] Chiou TT, Bonhomme B, Jin H, Miralles CP, Xiao H, Fu Z, Harvey RJ, Harvey K, Vicini S, De Blas AL. Differential regulation of the postsynaptic clustering of γ-aminobutyric acid type A (GABAA) receptors by collybistin isoforms. J Biol Chem. 2011; 286:22456–68. 10.1074/jbc.M111.236190 21540179 PMC3121391

[46] Dejanovic B, Schwarz G. Neuronal nitric oxide synthase-dependent S-nitrosylation of gephyrin regulates gephyrin clustering at GABAergic synapses. J Neurosci. 2014; 34:7763–68. 10.1523/JNEUROSCI.0531-14.2014 24899700 PMC6608267

[47] Prior P, Schmitt B, Grenningloh G, Pribilla I, Multhaup G, Beyreuther K, Maulet Y, Werner P, Langosch D, Kirsch J, Betz H. Primary structure and alternative splice variants of gephyrin, a putative glycine receptor-tubulin linker protein. Neuron. 1992; 8:1161–70. 10.1016/0896-6273(92)90136-2 1319186

[48] Sola M, Bavro VN, Timmins J, Franz T, Ricard-Blum S, Schoehn G, Ruigrok RW, Paarmann I, Saiyed T, O’Sullivan GA, Schmitt B, Betz H, Weissenhorn W. Structural basis of dynamic glycine receptor clustering by gephyrin. EMBO J. 2004; 23:2510–19. 10.1038/sj.emboj.7600256 15201864 PMC449768

[49] Ward KM, Aletras AH, Balaban RS. A new class of contrast agents for MRI based on proton chemical exchange dependent saturation transfer (CEST). J Magn Reson. 2000; 143:79–87. 10.1006/jmre.1999.1956 10698648

[50] Zaiss M, Bachert P. Chemical exchange saturation transfer (CEST) and MR Z-spectroscopy in vivo: a review of theoretical approaches and methods. Phys Med Biol. 2013; 58:R221–69. 10.1088/0031-9155/58/22/R221 24201125

[51] van Zijl PC, Lam WW, Xu J, Knutsson L, Stanisz GJ. Magnetization Transfer Contrast and Chemical Exchange Saturation Transfer MRI. Features and analysis of the field-dependent saturation spectrum. Neuroimage. 2018; 168:222–41. 10.1016/j.neuroimage.2017.04.045 28435103 PMC5650949

[52] Zhou J, Payen JF, Wilson DA, Traystman RJ, van Zijl PC. Using the amide proton signals of intracellular proteins and peptides to detect pH effects in MRI. Nat Med. 2003; 9:1085–90. 10.1038/nm907 12872167

[53] Kehagia AA, Barker RA, Robbins TW. Neuropsychological and clinical heterogeneity of cognitive impairment and dementia in patients with Parkinson’s disease. Lancet Neurol. 2010; 9:1200–13. 10.1016/S1474-4422(10)70212-X 20880750

[54] Owen AM, James M, Leigh PN, Summers BA, Marsden CD, Quinn NP, Lange KW, Robbins TW. Fronto-striatal cognitive deficits at different stages of Parkinson’s disease. Brain. 1992; 115:1727–51. 10.1093/brain/115.6.1727 1486458

[55] Taylor AE, Saint-Cyr JA, Lang AE. Frontal lobe dysfunction in Parkinson’s disease. The cortical focus of neostriatal outflow. Brain. 1986; 109:845–83. 10.1093/brain/109.5.845 3779372

[56] Maiti P, Gregg LC, McDonald MP. MPTP-induced executive dysfunction is associated with altered prefrontal serotonergic function. Behav Brain Res. 2016; 298:192–201. 10.1016/j.bbr.2015.09.014 26393431 PMC4803113

[57] Christopher L, Marras C, Duff-Canning S, Koshimori Y, Chen R, Boileau I, Segura B, Monchi O, Lang AE, Rusjan P, Houle S, Strafella AP. Combined insular and striatal dopamine dysfunction are associated with executive deficits in Parkinson’s disease with mild cognitive impairment. Brain. 2014; 137:565–75. 10.1093/brain/awt337 24334314 PMC4454524

[58] Pflibsen L, Stang KA, Sconce MD, Wilson VB, Hood RL, Meshul CK, Mitchell SH. Executive function deficits and glutamatergic protein alterations in a progressive 1-methyl-4-phenyl-1,2,3,6-tetrahydropyridine mouse model of Parkinson’s disease. J Neurosci Res. 2015; 93:1849–64. 10.1002/jnr.23638 26332770 PMC4618105

[59] Durand E, Petit O, Tremblay L, Zimmer C, Sgambato-Faure V, Chassain C, Laurent M, Pereira B, Silberberg C, Durif F. Social behavioral changes in MPTP-treated monkey model of Parkinson’s disease. Front Behav Neurosci. 2015; 9:42. 10.3389/fnbeh.2015.00042 25767440 PMC4341564

[60] Howard MW, Rizzuto DS, Caplan JB, Madsen JR, Lisman J, Aschenbrenner-Scheibe R, Schulze-Bonhage A, Kahana MJ. Gamma oscillations correlate with working memory load in humans. Cereb Cortex. 2003; 13:1369–74. 10.1093/cercor/bhg084 14615302

[61] Tawab MA, Bahr U, Karas M, Wurglics M, Schubert-Zsilavecz M. Degradation of ginsenosides in humans after oral administration. Drug Metab Dispos. 2003; 31:1065–71. 10.1124/dmd.31.8.1065 12867496

[62] Choi HS, Kim SY, Park Y, Jung EY, Suh HJ. Enzymatic transformation of ginsenosides in Korean Red Ginseng (Panax ginseng Meyer) extract prepared by Spezyme and Optidex. J Ginseng Res. 2014; 38:264–69. 10.1016/j.jgr.2014.05.005 25379006 PMC4213822

[63] Bae EA, Park SY, Kim DH. Constitutive beta-glucosidases hydrolyzing ginsenoside Rb1 and Rb2 from human intestinal bacteria. Biol Pharm Bull. 2000; 23:1481–85. 10.1248/bpb.23.1481 11145182

[64] Chen Q, Wells MM, Arjunan P, Tillman TS, Cohen AE, Xu Y, Tang P. Structural basis of neurosteroid anesthetic action on GABA_A_ receptors. Nat Commun. 2018; 9:3972. 10.1038/s41467-018-06361-4 30266951 PMC6162318

[65] Geng Y, Bush M, Mosyak L, Wang F, Fan QR. Structural mechanism of ligand activation in human GABA(B) receptor. Nature. 2013; 504:254–59. 10.1038/nature12725 24305054 PMC3865065

[66] Karunakaran S, Saeed U, Mishra M, Valli RK, Joshi SD, Meka DP, Seth P, Ravindranath V. Selective activation of p38 mitogen-activated protein kinase in dopaminergic neurons of substantia nigra leads to nuclear translocation of p53 in 1-methyl-4-phenyl-1,2,3,6-tetrahydropyridine-treated mice. J Neurosci. 2008; 28:12500–09. 10.1523/JNEUROSCI.4511-08.2008 19020042 PMC6671725

[67] Jackson-Lewis V, Przedborski S. Protocol for the MPTP mouse model of Parkinson’s disease. Nat Protoc. 2007; 2:141–51. 10.1038/nprot.2006.342 17401348

[68] Zhang Y, He X, Meng X, Wu X, Tong H, Zhang X, Qu S. Regulation of glutamate transporter trafficking by Nedd4-2 in a Parkinson’s disease model. Cell Death Dis. 2017; 8:e2574. 10.1038/cddis.2016.454 28151476 PMC5386455

[69] Amende I, Kale A, McCue S, Glazier S, Morgan JP, Hampton TG. Gait dynamics in mouse models of Parkinson’s disease and Huntington’s disease. J Neuroeng Rehabil. 2005; 2:20. 10.1186/1743-0003-2-20 16042805 PMC1201165

[70] Samantaray S, Knaryan VH, Shields DC, Cox AA, Haque A, Banik NL. Inhibition of Calpain Activation Protects MPTP-Induced Nigral and Spinal Cord Neurodegeneration, Reduces Inflammation, and Improves Gait Dynamics in Mice. Mol Neurobiol. 2015; 52:1054–66. 10.1007/s12035-015-9255-6 26108182 PMC4558265

[71] Spowart-Manning L, van der Staay FJ. The T-maze continuous alternation task for assessing the effects of putative cognition enhancers in the mouse. Behav Brain Res. 2004; 151:37–46. 10.1016/j.bbr.2003.08.004 15084419

[72] Castro AA, Wiemes BP, Matheus FC, Lapa FR, Viola GG, Santos AR, Tasca CI, Prediger RD. Atorvastatin improves cognitive, emotional and motor impairments induced by intranasal 1-methyl-4-phenyl-1,2,3,6-tetrahydropyridine (MPTP) administration in rats, an experimental model of Parkinson’s disease. Brain Res. 2013; 1513:103–16. 10.1016/j.brainres.2013.03.029 23548600

[73] Kang J, Shin JW, Kim YR, Swanberg KM, Kim Y, Bae JR, Kim YK, Lee J, Kim SY, Sohn NW, Maeng S. Nobiletin improves emotional and novelty recognition memory but not spatial referential memory. J Nat Med. 2017; 71:181–89. 10.1007/s11418-016-1047-4 27830412

[74] Ennaceur A, Delacour J. A new one-trial test for neurobiological studies of memory in rats. 1: behavioral data. Behav Brain Res. 1988; 31:47–59. 10.1016/0166-4328(88)90157-X 3228475

[75] Yan G, Zhang T, Dai Z, Yi M, Jia Y, Nie T, Zhang H, Xiao G, Wu R. A Potential Magnetic Resonance Imaging Technique Based on Chemical Exchange Saturation Transfer for In Vivo γ-Aminobutyric Acid Imaging. PLoS One. 2016; 11:e0163765. 10.1371/journal.pone.0163765 27711138 PMC5053432

[76] Müller-Lutz A, Matuschke F, Schleich C, Wickrath F, Boos J, Schmitt B, Wittsack HJ. Improvement of water saturation shift referencing by sequence and analysis optimization to enhance chemical exchange saturation transfer imaging. Magn Reson Imaging. 2016; 34:771–78. 10.1016/j.mri.2016.03.013 26988704

[77] Kim M, Gillen J, Landman BA, Zhou J, van Zijl PC. Water saturation shift referencing (WASSR) for chemical exchange saturation transfer (CEST) experiments. Magn Reson Med. 2009; 61:1441–50. 10.1002/mrm.21873 19358232 PMC2860191

[78] Zhang Y, He X, Wu X, Lei M, Wei Z, Zhang X, Wen L, Xu P, Li S, Qu S. Rapamycin upregulates glutamate transporter and IL-6 expression in astrocytes in a mouse model of Parkinson’s disease. Cell Death Dis. 2017; 8:e2611. 10.1038/cddis.2016.491 28182002 PMC5386462

[79] Qu S, Meng X, Liu Y, Zhang X, Zhang Y. Ginsenoside Rb1 prevents MPTP-induced changes in hippocampal memory via regulation of the α-synuclein/PSD-95 pathway. Aging (Albany NY). 2019; 11:1934–64. 10.18632/aging.101884 30958793 PMC6503885

