# Supplementary material for: Ginsenoside Rb1 regulates prefrontal cortical GABAergic transmission in MPTP-treated mice
Source: Aging (Albany NY). 2019 Jul 17;11(14):5008–34. doi: 10.18632/aging.102095 (PMC6682523; doi:10.18632/aging.102095)
Supplement: Supplementary Figures [file aging-11-102095-s001.pdf]

SUPPLEMENTARY FIGURES

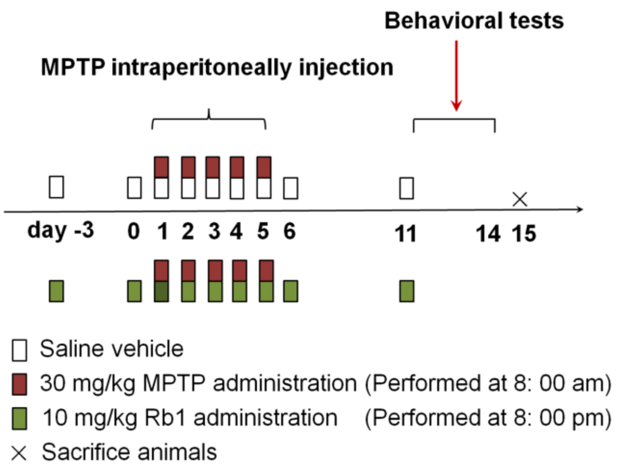

Supplementary Figure 1. Experimental time-line. Saline vehicle mice were intraperitoneally injected with vehicle (saline) once from day -3 to day 0 or day 6 to day 11, and twice from day 1 to day 5. MPTP mice were intraperitoneally injected with vehicle (saline) once from day -3 to day 0 or day 6 to day 11, and intraperitoneally injected with MPTP and saline from day 1 to day 5. Rb1 treatment mice were intraperitoneally injected with 10 mg/kg Rb1 once from day -3 to day 11, and intraperitoneally injected with MPTP from day 1 to day 5. All PD model animals were generated by administration of MPTP intraperitoneally for 5 consecutive days at a dose of 30 mg/kg free base (MPTP-HCl) in saline. The time interval between MPTP and Rb1 injections was more than 12 h (MPTP was given at 8:00 am and Rb1 was given at 8:00 pm). One day after the last Rb1/saline injection, behavioral tests were performed and the animals were sacrificed by isoflurane anesthesia for tissue collection and electrophysiological recording.

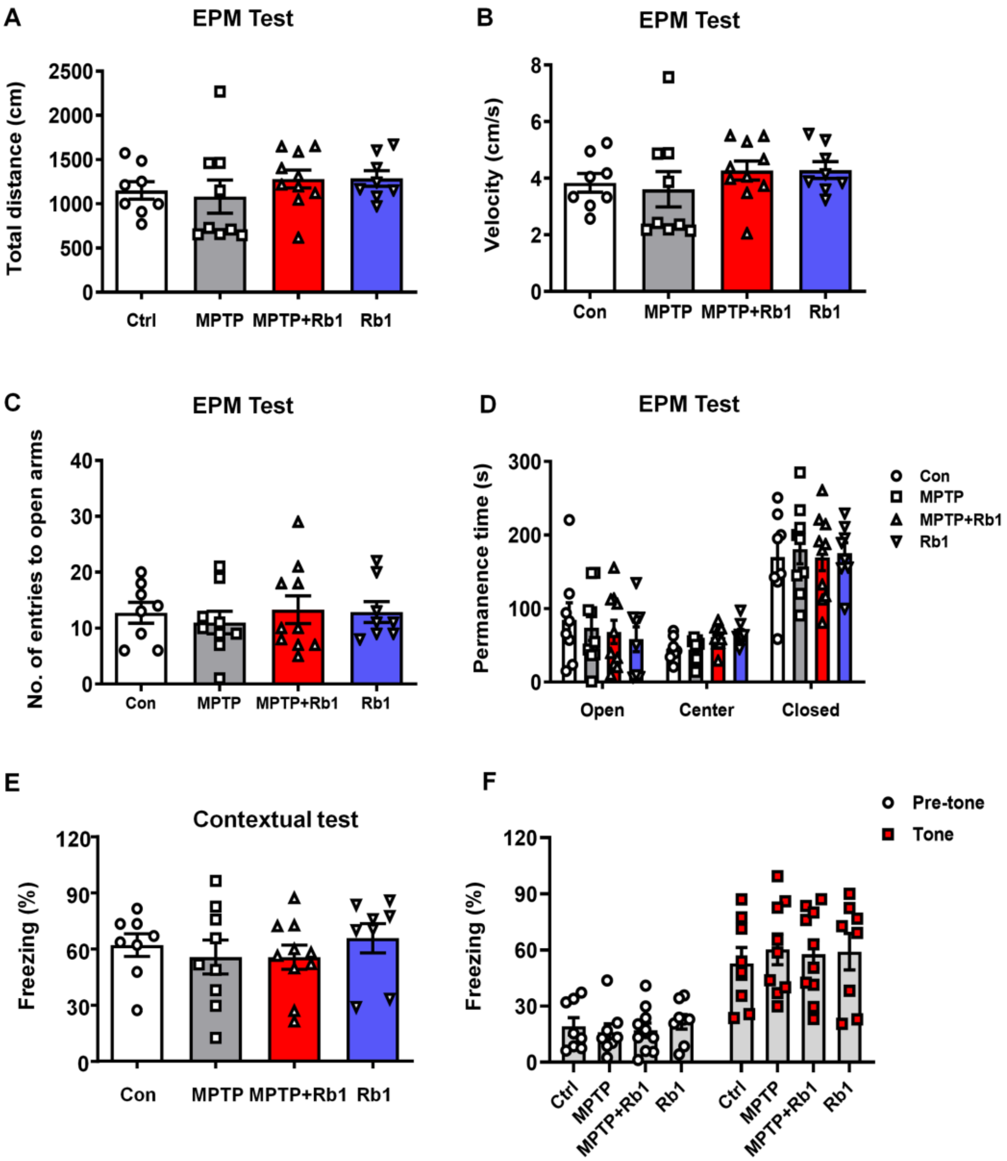

Supplementary Figure 2. Effect of Rb1 on the elevated plus maze (EPM) and fear conditioning test in MPTP-treated mice. (**A-D**) Total travelled distance, movement speed, number of entries to the center, and the time spent in the open-, center- and closed-field after Rb1 administration in MPTP-treated mice were examined by EPM test. Contextual freezing behaviors (**E**), pre-tone freezing behaviors or fear expression (**F**) after Rb1 administration in MPTP-treated mice were examined by fear conditioning test. n = 8 in control group, n = 9 in MPTP group, n = 10 in MPTP+Rb1 group and n = 8 in Rb1 group. Results are expressed as the mean ± SEM. Statistical significance was determined by one-way ANOVA and Bonferroni test as *post hoc* comparisons.

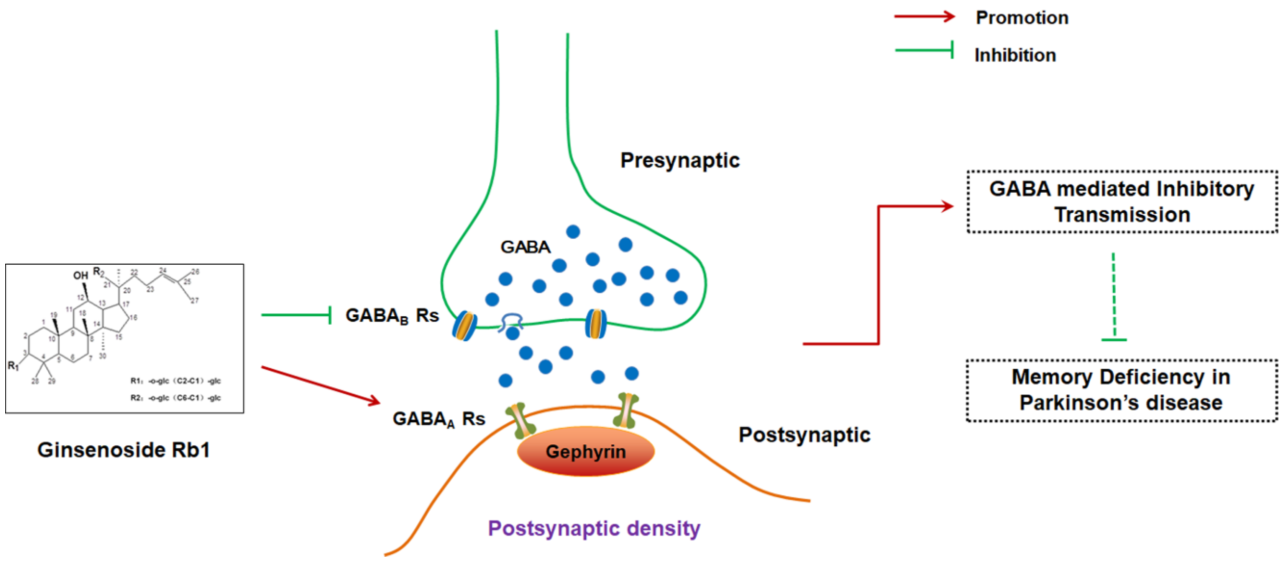

Supplementary Figure 3. Schematic models showing the neuroprotective mechanism of Rb1 in the PD models. Ginsenoside Rb1 can bind with GABA$_A$Rα1 and increase its expression may through postsynaptic anchored gephyrin in the PFC of MPTP mice model. In addition, Rb1 may suppress presynaptic GABA$_B$R1 to enhance GABA release. Taken together, Rb1 can promote prefrontal cortical GABA content and GABAergic transmission in MPTP mice model, and this neuroprotection may account for Rb1's amelioration in PD-associated cognitive deficits.
